# A tungsten-specific maturation pathway governs cofactor assembly of a CO_2_-reducing formate dehydrogenase in *Methylorubrum extorquens*

**DOI:** 10.1016/j.jbc.2026.111483

**Published:** 2026-04-22

**Authors:** Uyen Thu Phan, Yong Hwan Kim

**Affiliations:** 1 School of Energy and Chemical Engineering, Ulsan National Institute of Science and Technology, Ulsan, Republic of Korea; 2 Graduate School of Carbon Neutrality, Ulsan National Institute of Science and Technology, Ulsan, Republic of Korea

**Keywords:** cofactor biosynthesis, formate dehydrogenase (MeFDH1), enzyme mechanism, *Methylorubrum extorquens*, MoeA1, MobB, metalloenzyme, protein-protein interaction, tungsten cofactor (W-bis-MGD)

## Abstract

Tungsten-dependent formate dehydrogenases catalyze the reversible interconversion of carbon dioxide and formate and play key roles in microbial redox metabolism, yet the molecular basis for selective tungsten cofactor assembly in aerobic bacteria remains poorly understood. Here, we define a dedicated tungsten-specific maturation pathway that governs cofactor assembly and delivery to the carbon dioxide-reducing formate dehydrogenase MeFDH1 in *Methylorubrum extorquens* AM1. Targeted genetic deletions identified *moeA1*, *mobB*, and *fdhD* as essential for MeFDH1 activity and tungsten incorporation. Protein-protein interaction and structural modeling analyses place MoeA1 at the center of this pathway, where a MoeA1-MobB platform mediates tungstate insertion into the pterin scaffold, followed by MobA-dependent guanylylation to generate W-bis-molybdopterin guanine dinucleotide and FdhD-catalyzed sulfuration and cofactor delivery to MeFDH1. Mutagenesis of residues within the predicted metal-binding pocket and metal competition experiments further indicate that the electrostatic environment of the MoeA1 biases cofactor assembly toward tungsten. Together, our findings establish a tungsten-selective cofactor assembly pathway in an aerobic bacterium and offer strategies for engineering tungsten-dependent redox enzymes for applications in microbial carbon fixation and biocatalysis.

Formate dehydrogenases (FDHs) are molybdopterin-containing metalloenzymes that catalyze the reversible interconversion of formate and carbon dioxide (CO_2_). They are central to microbial one-carbon (C1) metabolism and have attracted interest as biocatalysts for carbon capture and synthetic formate production (1, 2, 3, 4). While most FDHs are molybdenum (Mo)-dependent, several organisms—including hyperthermophiles and methylotrophs—express tungsten (W)-containing FDHs with distinct redox properties, often favoring CO_2_ reduction under reducing conditions (5, 6, 7, 8, 9). These W-dependent enzymes offer enhanced thermodynamic potential and oxygen tolerance, making them attractive for CO_2_-to-formate electroconversion and industrial biocatalysis (10, 11, 12).

Among them, the FDH from *Methylorubrum extorquens* (MeFDH1) has emerged as a promising biocatalyst due to its involvement in the low-potential reduction of CO_2_ (13, 14, 15). This heterodimeric (αβ) enzyme contains a W-bis-molybdopterin guanine dinucleotide (W-bis-MGD) cofactor and iron-sulfur clusters yet lacks selenocysteine and remains active under aerobic conditions (5, 16). Owing to its robust activity, oxygen tolerance, selectivity and scalable potential, MeFDH1 is being actively developed for sustainable CO_2_ utilization in the production of biofuels, chemicals, and energy storage systems (13, 14, 15). However, scaling up the production of MeFDH1 remains a major technical challenge. A central barrier is the biosynthesis and proper incorporation of its essential W-based cofactor (17, 18). This cofactor is indispensable for catalysis and characteristic of W-dependent FDHs.

Molybdenum cofactor (Moco) and tungsten cofactor (Wco) utilize the same pyranopterin cofactor framework (19, 20). The core biosynthetic pathway that produces the molybdopterin (MPT) scaffold is highly conserved across species, proceeding through three major stages: (i) conversion of GTP to cyclic pyranopterin monophosphate, (ii) conversion of cyclic pyranopterin monophosphate to MPT, and (iii) metal insertion into MPT to generate the mature cofactor (19, 20). In W-utilizing organisms, the early reactions are identical to those in Mo biosynthesis, producing a common adenylylated MPT intermediate, with metal specificity imposed only at the final insertion step (21). As a result, Moco and Wco differ primarily in the identity of the incorporated metal (17).

Because molybdate (MoO_4_^2-^) and tungstate (WO_4_^2-^) are chemically analogous, a longstanding question is how cells discriminate between these metals during cofactor insertion and subsequently channel the metal-loaded cofactor to the correct apoenzyme. Although the upstream reactions that construct the pyranopterin scaffold are shared, the late stage—metal insertion and conversion of metalated MPT into the dinucleotide forms (Mo-bis-MGD and W-bis-WGD)—appears to be the critical control point that defines Moco *versus* Wco biosynthesis. Emerging evidence suggests that organisms producing both Mo- and W-enzymes have evolved metal-specific biosynthetic modules composed of dedicated enzymes specialized for Mo or W uptake and insertion, and maturation factors (such as FdhD for FDHs) that prevent crosstalk and ensure precise cofactor delivery (17). Nevertheless, the molecular basis of this metal discrimination and hand-off remains incompletely understood. In particular, the identity and mechanism of the enzyme(s) that insert tungstate into adenylylated MPT have not been conclusively established (17, 18).

In this study, we investigated the specialized W maturation pathway responsible for MeFDH1 activity using *M*. *extorquens* as a model system. This organism encodes both W- and Mo-FDHs (22, 23), suggesting the coexistence of distinct Moco and Wco biosynthetic routes. By identifying the factors that enable W-containing MeFDH1 maturation and visualizing cofactor transfer complexes, we aim to illuminate how *M*. *extorquens* achieves W selectivity. Such findings will not only address critical gaps in the understanding of W-FDH maturation process but also provide a framework for engineering robust FDHs for biotechnological applications in carbon capture, renewable energy, and synthetic metabolism.

## Results

### MoeA1, MobB, and FdhD are indispensable for MeFDH1 maturation

**Figure 1:**
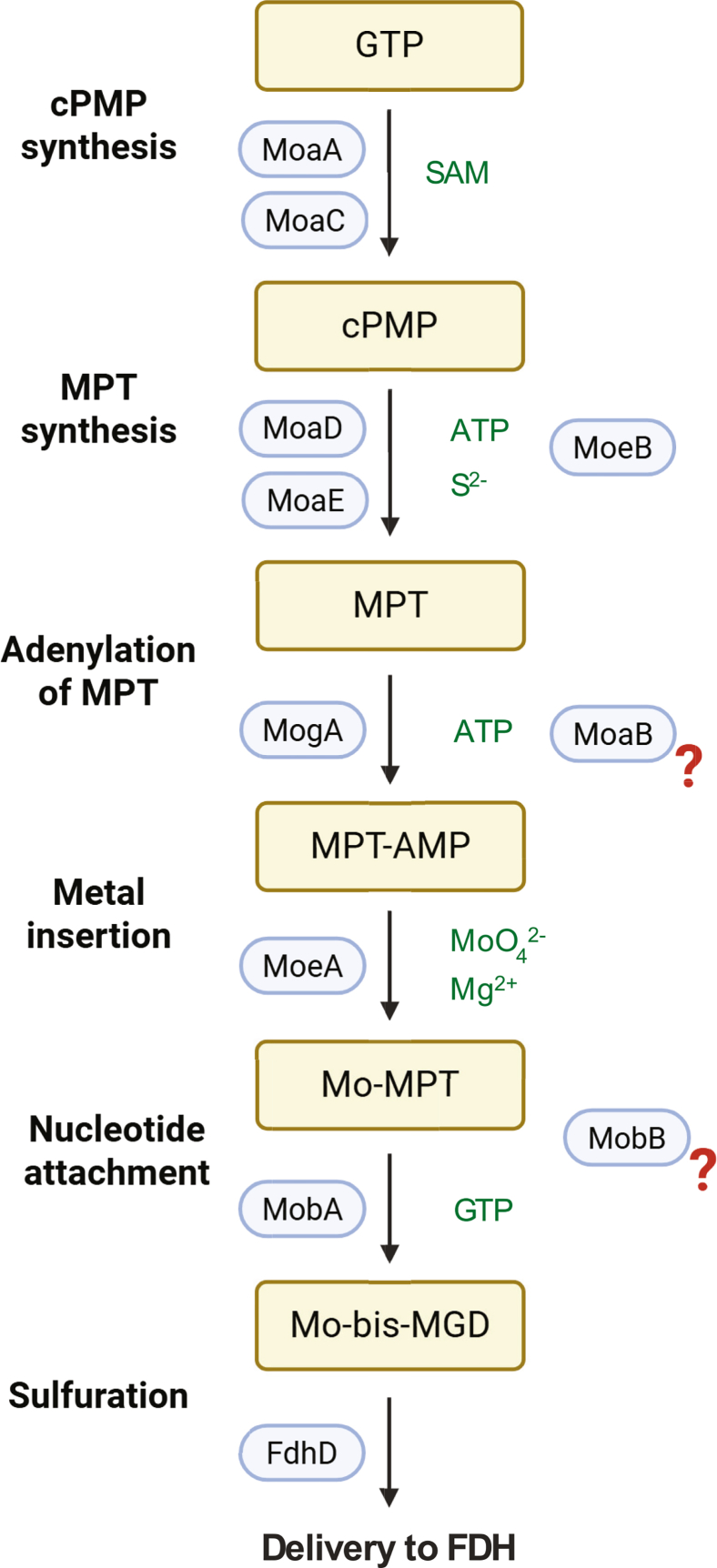
**Schematic overview of molybdenum cofactor (Mo-bis-MGD) biosynthesis and delivery to formate dehydrogenase**. The biosynthetic pathway initiates from GTP, which is converted to cyclic pyranopterin monophosphate by MoaA and MoaC using S-adenosyl-L-methionine (SAM). The subsequent sulfur insertion and formation of molybdopterin (MPT) require MoaD, MoaE, and the sulfurase MoeB in an ATP- and sulfide-dependent process. MPT is then adenylated by MogA in an ATP-dependent step to yield adenylylated MPT, followed by metal insertion catalyzed by MoeA in the presence of molybdate (MoO_4_^2-^) and Mg^2+^, generating Mo-MPT. The final step involves MobA, which attaches two molecules of GMP to form Mo-bis-MGD in a GTP-dependent reaction. The completed cofactor is delivered to the target molybdoenzyme by FdhD. The roles of MoaB and MobB in assisting MogA, MoeA, or MobA steps remain uncertain and indicated by red question marks.

To resolve how *M*. *extorquens* assembles the Wco (W-bis-MGD) for its CO_2_-reducing formate dehydrogenase (FDH) MeFDH1, we first benchmarked against the canonical Moco pathway in *Escherichia coli* (Fig. 1) and identified orthologous genes in the *M*. *extorquens* genome (Table 1). Because the core biosynthetic framework is conserved, divergence between Moco and Wco pathways is expected during the late maturation stages, where metal specificity is established and transmitted to the apoenzyme (17, 18, 19, 24). Although late-stage Moco maturation is well characterized, the corresponding W-specific reactions remain poorly defined, particularly the roles of MoaB and MobB across lineages (18, 25). In addition, *M*. *extorquens* encodes paralogous MoeA and MobA proteins whose functional assignments are unresolved (Table 1). These gaps motivate a focused analysis of the terminal Wco steps governing how tungstate is selected over molybdate, inserted into the MPT scaffold, and converted to W-bis-MGD. Because terminal sulfuration and delivery are critical checkpoints for FDH activation, we also investigated the FDH-specific sulfurtransferase FdhD, thereby centering our analyses on the metal-choice and hand-off segment most relevant to MeFDH1 (26).

**Table 1:** The presence of Moco biosynthetic proteins in *Methylorubrumextorquens*

Function of step	Proteins in *E*. *coli*	Proteins in *M*. *extorquens*	UniProt/ locus in *M*. *extorquens*	% Identity vs *E*. *coli*
cPMP synthesis	MoaA	MoaA	C5AUI6/ MexAM1_META1p2843	32.2
	MoaC	MoaC	C5AU27/ MexAM1_META1p5122	52.8
MPT synthesis	MoaD	MoaD	C5AVZ3/ MexAM1_META1p3081	33.8
	MoaE	MoaE	C5AZC7/ MexAM1_META1p1399	47.6
	MoeB	MoeB	C5B038/ MexAM1_META1p1526	42.6
Adenylation of MPT	MogA	Mog	C5ASG5/ MexAM1_META1p2636	50.3
Metal insertion	MoeA	MoeA1	C5AVW2/ MexAM1_META1p3050	40.6
		MoeA2	C5B450/ MexAM1_META2p0368	37.4
		MoeA3	C5AVW0/ MexAM1_META1p3048	36.3
		MoeA4	C5AU28/ MexAM1_META1p5123	34.9
		MoeA5	C5AVV9/ MexAM1_META1p3047	26.7
Nucleotide attachment	MobA	MobA	C5ATS0/ MexAM1_META1p5015	35.3
		MobA-like	C5AWC1/ MexAM1_META1p0828	25.3
Sulfuration/delivery to FDH	FdhD	FdhD	C5AYB3/ MexAM1_META1p3475	36.1
Not determined	MoaB	MoaB	C5AVN0/ MexAM1_META1p0772	47.1
	MobB	MobB	C5AVW1/ MexAM1_META1p3049	40.2

Targeted deletions were introduced across the late-acting genes, corresponding to the stages of metal insertion, guanylylation, and cofactor delivery. All candidates were successfully disrupted except *mobA* and *moaB*, for which repeated allelic-replacement attempts failed to yield viable mutants, precluding direct knockout phenotyping and suggesting essentiality under our conditions (Table 2). Among the viable mutants, loss of *moeA1*, *mobB*, or *fdhD* abolished MeFDH1 activity and eliminated W incorporation while leaving growth and overall protein abundance largely unchanged (Fig. S1), consistent with a failure to assemble or retain a metalated cofactor. By contrast, deletions of *moeA2–moeA5*, *mog*, or the *mobA-*like locus had minimal impact on MeFDH1 activity and preserved W occupancy. Together, these findings identify *moeA1*, *mobB*, and *fdhD* as indispensable for MeFDH1 maturation and establish MoeA1 as the dedicated tungstate insertase in *M*. *extorquens*.

**Table 2:** MeFDH1 activity and metal occupancy in *Methylorubrumextorquens* deletion strains

Strain	MeFDH1 specific activity (U mg^-1^)	Mo content (mol Mo mol^-1^ MeFDH1)	W content (mol W mol^-1^ MeFDH1)
WT	50 ± 4	ND	0.48 ± 0.03
Essential for activity			
Δ*moeA1*	ND	ND	ND
Δ*mobB*	ND	ND	ND
Δ*fdhD*	ND	ND	ND
Dispensable			
Δ*moeA2*	51 ± 5	ND	0.41 ± 0.13
Δ*moeA3*	41 ± 4	ND	0.33 ± 0.10
Δ*moeA4*	42 ± 4	ND	0.40 ± 0.03
Δ*moeA5*	40 ± 2	ND	0.37 ± 0.05
Δ*mog*	51 ± 2	ND	0.53 ± 0.02
Δ*mobA*-like	40 ± 2	ND	0.30 ± 0.10
Likely essential			
Δ*moaB*	NA	NA	NA
Δ*mobA*	NA	NA	NA

MeFDH1 purified from WT and the indicated gene-deletion strains was assayed for CO_2_-reducing activity using reduced ethyl viologen (EV) as an artificial electron donor. Specific activities are reported as units per milligram of MeFDH1 (U mg^-1^; mean ± SD, n = 3). Tungsten (W) and molybdenum (Mo) contents of the purified enzyme were quantified by ICP–OES and normalized to the molar concentration of MeFDH1. Functional category labels summarize the phenotypic outcome of each deletion: Essential for activity (loss of MeFDH1 activity and W metalation), Dispensable (activity and W incorporation comparable to WT), and Likely essential (no viable knockout obtained after repeated allelic-replacement attempts). ND, below the limit of detection; NA, no viable knockout recovered.

Plasmid complementation restored MeFDH1 activity in parallel with W content in Δ*moeA1*-C, Δ*mobB*-C, and Δ*fdhD*-C strains, confirming the direct involvement of these genes for productive Wco assembly and delivery (Table S1). Notably, *fdhD* overexpression elevated specific activity and W accumulation by approximately 30%, indicating that FdhD-mediated sulfido insertion and cofactor transfer can represent a kinetic bottleneck in generating catalytically competent MeFDH1.

Metal analysis of purified MeFDH1 demonstrated a linear relationship between W content and specific activity across all strains (Fig. S2), reinforcing that catalytic competence directly depends on successful W-bis-MGD insertion. Mo was consistently below detection limits, confirming strict W selectivity of the holoenzyme. The restoration of both activity and W occupancy upon genetic complementation further supports that loss of activity in deletion strains originates from defective metalation rather than protein instability or expression defects.

### Distinct Mo and W cofactor biosynthetic routes

**Figure 2:**
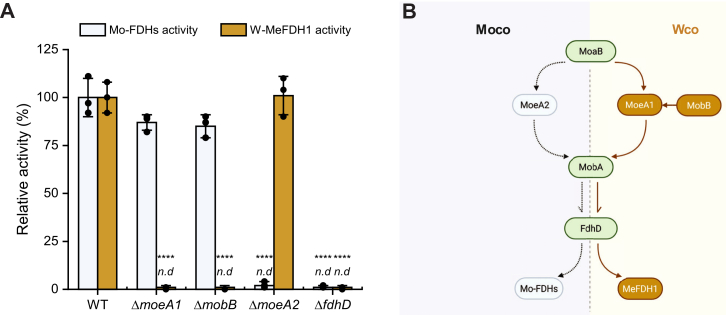
**Metal-cofactor specialization for molybdenum- and tungsten-dependent formate dehydrogenase in *Methylorubrum extorquens*.***A*, relative activities of molybdenum-dependent FDHs (Mo-FDHs, *gray*) measured in molybdate-grown cultures and the tungsten-dependent MeFDH1 (*brown*) measured under tungsten-supplemented conditions in WT and indicated deletion strains. Activities are normalized to WT levels. Data represent mean ± SD from three independent biological replicates (n = 3). Statistical significance was determined using a two-tailed Student’s *t* test with comparisons to WT. *Asterisks* denote statistically significant differences (∗∗∗∗, *p* < 0.0001); only significant comparisons are shown. n.d., not detectable. *B*, schematic model illustrating the genetically and mechanistically distinct late-stage cofactor maturation branches. The tungsten-dependent pathway comprises MoaB, MoeA1, MobB, MobA, and FdhD and directs Wco assembly and delivery to MeFDH1, whereas MoeA2 supports a parallel molybdenum-dependent pathway that matures Mo-FDHs. Node colors denote functional categories: W-biased components (*brown*), shared maturation factors (*green*), and Mo-branch proteins (*gray*). Wco, tungsten cofactor.

When cells were cultivated with molybdate in the absence of added tungstate, cellular Mo-FDHs activity (gray bar in Figure 2*A*) was retained in Δ*moeA1* and Δ*mobB* strains but abolished in Δ*fdhD* and Δ*moeA2* backgrounds. Because MeFDH1 is deleted from the chromosome in all strains used for Mo-FDH measurements, these activities exclusively reflect endogenous Mo-FDHs. The strict dependence of Mo-FDHs activity on MoeA2, but not on MoeA1 or MobB, under these conditions provides independent support for MoeA2 functioning as the principal insertase in the Mo-specific maturation pathway, whereas MoeA1 together with MobB defines a W-specific maturation route. In contrast, FdhD is required for both activities, consistent with its conserved role in terminal sulfuration and targeted cofactor delivery (26).

Under same molybdate growth conditions, Δ*mog* and Δ*mobA*-like mutants displayed Mo-FDH activities comparable to wild-type, implying that neither Mog nor the MobA-like paralog is rate-limiting for metal-dependent maturation (Fig. S3). Conversely, repeated attempts to generate Δ*moaB* or Δ*mobA* mutants were unsuccessful, suggesting that these genes play essential roles in the maturation pathway. Because deletion mutants could not be obtained, we describe *moaB* and *mobA* as likely essential under the tested conditions, consistent with their central roles in the pterin adenylylation and guanylylation reactions required for formation of the dinucleotide cofactor MGD shared by both Mo- and W-dependent pathways and supported by prior genetic and biochemical studies (17, 21, 27, 28, 29, 30, 31, 32).

Together, these findings support a model in which *M*. *extorquens* employs two genetically distinct Moco and Wco maturation branches that diverge after MPT adenylation (Fig. 2*B*), with the W branch dedicated to assembly of the W-bis-MGD cofactor required for MeFDH1.

### Interaction network underlying Wco assembly

Although genetic and biochemical analyses identified the factors essential for MeFDH1 activation, these approaches did not resolve how the maturation machinery is physically organized. In *E*. *coli*, the terminal reactions of Moco biogenesis are mediated through protein-protein interaction networks that couple late biosynthetic enzymes with enzyme-specific chaperones and apoenzymes, forming coordinated maturation assemblies rather than freely diffusing components (33). Given the close conservation of late-stage steps, we hypothesized that Wco assembly in *M*. *extorquens* likewise depends on analogous interaction network linking the late-acting biosynthetic factors to the MeFDH1 apoenzyme.

**Figure 3:**
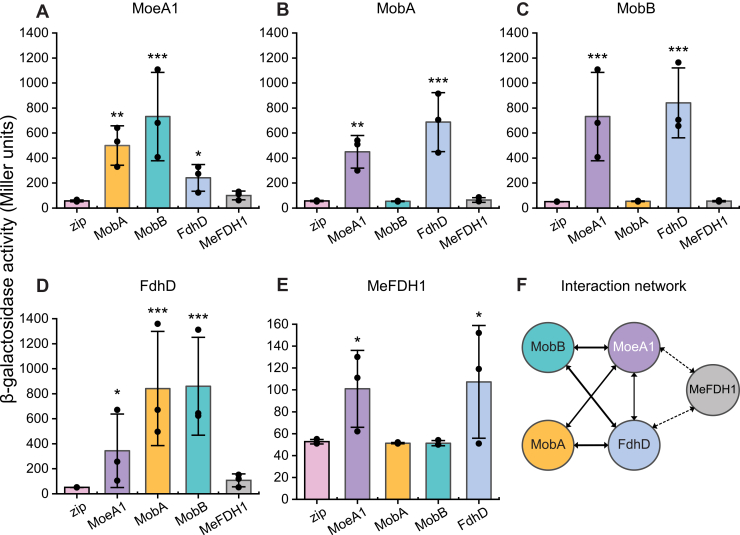
**BACTH-defined interaction network governing late-stage tungsten cofactor maturation and delivery**. *A–E*, pairwise bacterial adenylate cyclase two-hybrid (BACTH) assays were performed in *E*. *coli* BTH101 using T25/T18 fusion constructs of *M*. *extorquens* MoeA1, MobA, MobB, FdhD, and MeFDH1. β-galactosidase activity is reported as Miller units (mean ± SD) from three independent biological replicates (n = 3). Statistical significance relative to the negative control (zip) was assessed using a two-tailed Student’s *t* test. Significance is indicated as *p* < 0.05 (∗), *p* < 0.01 (∗∗), and *p* < 0.001 (∗∗∗); only statistically significant comparisons are shown. Signals exceeding background levels indicate protein-protein interaction, with higher MU values reflecting increased reporter activation but not necessarily proportional differences in binding affinity. *F*, schematic summary of the W-specific interaction network inferred from BACTH analysis. Edge thickness corresponds to statistical significance observed in panels *A*–*E* (thin, *p* < 0.05; medium, *p* < 0.01; thick, *p* < 0.001), and *dashed arrows* indicate *lower* but above-background reporter output. These representations reflect relative interaction strength under the assay conditions and do not imply quantitative binding affinity or interaction lifetime. Only interactions exceeding background levels are shown; pairwise combinations with near-background activity were omitted for clarity.

Comprehensive bacterial adenylate cyclase two-hybrid (BACTH) analysis delineated a network of PPIs among late-stage Wco maturation factors. All MoaB combinations exhibited activities near the zip negative-control baseline (Fig. S4), indicating no detectable pairwise BACTH interaction for MoaB under the conditions tested. In contrast, MoeA1 produced high BACTH reporter activity with both MobA and MobB, as well as intermediate activity with FdhD (Fig. 3, *A*–*D*), positioning MoeA1 as the central interaction hub. FdhD also showed above-background reporter activity with MobA and MobB (Fig. 3, *B*–*D*), consistent with engagement downstream of guanylylation during terminal sulfuration and delivery. Lower BACTH reporter activity detected with MeFDH1 (Fig. 3*E*) is compatible with a context-dependent interaction that may become more favorable after cofactor completion, in agreement with studies of nitrate reductase A, where productive assembly-apoenzyme contacts arise only after the mature cofactor has formed (34).

Because BACTH signals are influenced by both interaction-dependent cAMP reconstitution and fusion protein expression levels, these values should be interpreted as relative indicators of interaction propensity rather than quantitative measures of binding affinity. Collectively, these data define a W-specific interaction network centered on MoeA1 that integrates the terminal steps of Wco biosynthesis with its delivery to MeFDH1 (Fig. 3*F*).

### Structural predictions for Wco pathway components

**Figure 4:**
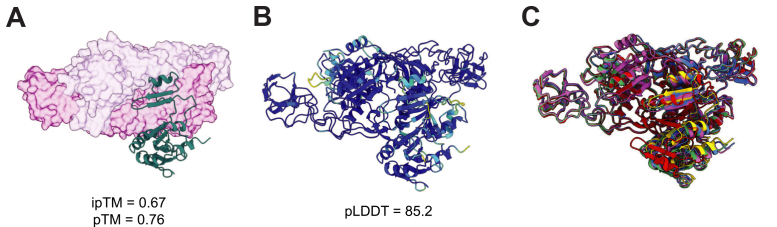
**Structural modeling of the MoeA1-MobB interaction**. *A*, AlphaFold 3-predicted MoeA1-MobB heterocomplex shown with MoeA1 rendered as a surface and MobB as a teal-colored cartoon. *B*, AF3 rank-1 MoeA1-MobB model displayed as a cartoon and colored by pLDDT confidence: *blue*, very high confidence (pLDDT > 90); *cyan*, confident (70 ≤ pLDDT ≤ 90); *yellow*, low confidence (50 ≤ pLDDT < 70); *orange*, very low confidence (pLDDT < 50). *C*, structural overlay of the top five AF3 MoeA1-MobB models, illustrating convergence of the predicted interaction interface. pLDDT, predicted local distance difference test.

To define the structural logic underlying the BACTH-derived interaction network, we applied AlphaFold 3 (AF3) multimer modeling to evaluate the stoichiometric and topological plausibility of all experimentally supported protein pairs (35). AF3 produced high overall confidence across all models, with mean predicted local distance difference test (pLDDT) values near or above 80 (Table S2), enabling comparison of interface confidence (ipTM) across binary combinations, which is summarized as a pairwise AF3 ranking score heat map (Fig. S5). Among all tested pairs, the MoeA1-MobB interaction yielded a robust prediction (ipTM = 0.67, mean pLDDT = 85.2; Figure 4, *A* and *B*), fully consistent with the strong BACTH signal. Overlay of the top-ranked models converged on a reproducible interface geometry (Fig. 4*C*), further supported by low predicted aligned error across the interaction surface (Fig. S6*A*), designating MoeA1-MobB as the core metal-insertion platform.

The FdhD-MeFDH1 delivery module reached moderate interface confidence (ipTM = 0.74; Fig. S7*A*), compatible with docking of FdhD onto the MeFDH1 α-subunit during sulfuration-coupled cofactor transfer. A MoeA1-MeFDH1 arrangement (Fig. S7*B*) placed MoeA1 adjacent to the apoenzyme interface, implying that the insertase remains engaged beyond metal insertion to guide delivery and explaining the failure of heterologous *E*. *coli* MoeA to activate MeFDH1 (16, 36).

**Figure 5:**
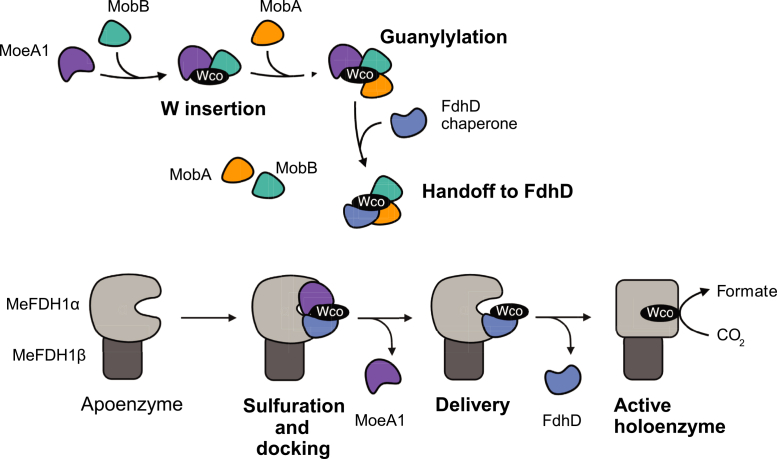
**Proposed stepwise channeling mechanism for late-stage tungsten cofactor (Wco) maturation and installation into MeFDH1**. Schematic model summarizing the sequential interactions inferred from genetic, BACTH, and AlphaFold 3 analyses. (*top*) Wco biosynthesis proceeds through a coordinated series of protein assemblies. MoeA1 and MobB form the core predicted platform that supports W-biased insertion. MobA is subsequently recruited in a handoff model to catalyze guanylylation, producing W-bis-molybdopterin guanine dinucleotide. The resulting intermediate is then transferred to the FdhD chaperone, which catalyzes terminal sulfuration to generate the mature Wco. (*bottom*) The sulfido-containing Wco-FdhD complex docks onto the MeFDH1 apoenzyme, completing cofactor delivery and formation of the active holoenzyme. MoeA1, MobA, and MobB act upstream in cofactor formation, whereas FdhD bridges the biosynthetic and enzymatic modules through a coupled sulfuration and docking event. Wco, tungsten cofactor.

All higher-order assemblies that included MobA and/or FdhD exhibited low-to-moderate interface confidence despite well-resolved monomer folds (Table S2), suggesting that these larger complexes are less well supported than the MoeA1-MobB pair. Overall, the AF3 metrics parallel the experimentally determined BACTH-derived network and are consistent with a stepwise handoff model rather than a preassembled megacomplex (Fig. 5).

### Molecular explanation for tungstate preference in MoeA1

**Figure 6:**
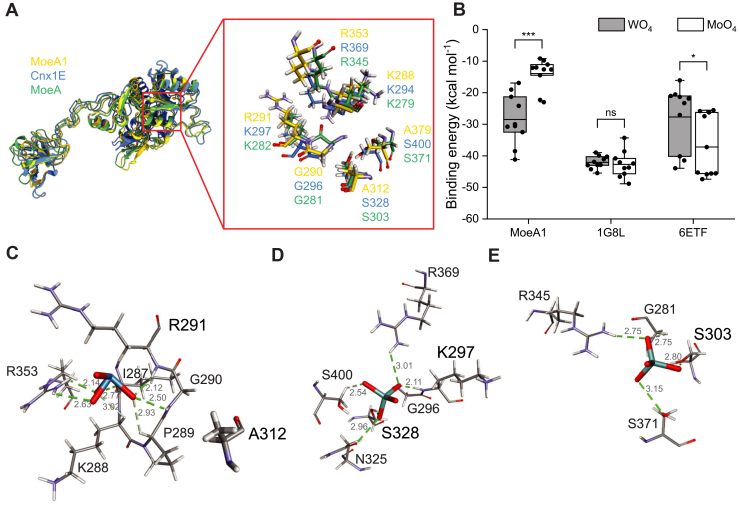
**Structural features and docking analyses underlying tungsten selectivity in MoeA1**. *A*, structural superposition of MoeA1 (*yellow*) with canonical Mo-insertases *Arabidopsis thaliana* Cnx1E (*blue*; PDB: 6ETF) and *E*. *coli* MoeA (*green*; PDB: 1G8L), highlighting differences in the oxyanion-binding pocket. Insets show key coordinating residues surrounding the bound oxyanion. *B*, docking-derived binding energies for WO_4_^2-^ and MoO_4_^2-^ in MoeA1, Cnx1E, and *E*. *coli* MoeA. Each *point* represents an individual docking pose (n = 10) obtained from a single simulation, with *bars* indicating mean ± SD. Statistical significance between WO_4_^2-^ and MoO_4_^2-^ binding within each protein was evaluated using a two-tailed Student’s *t* test. Significance is denoted as ns (not significant), *p* < 0.05 (∗), and *p* < 0.001 (∗∗∗). *C*, predicted tungstate-binding mode in MoeA1, showing an expanded hydrogen-bonding and electrostatic network. Hydrogen bonds between tungstate oxygens and coordinating residues are shown as dashed lines, with interatomic distances (Å) indicated. *D* and *E*, representative molybdate-binding configurations in Cnx1E (*D*) and *E*. *coli* MoeA (*E*), illustrating conserved interactions absent in MoeA1. *Dashed lines* indicate hydrogen bonds with distances (Å).

Given the central position of MoeA1 within the interaction network, we examined whether its metal-binding pocket is structurally tuned for selective tungstate recognition. Although MoeA1 preserves the overall fold of canonical Mo-insertases, including *Arabidopsis thaliana* Cnx1E (PDB 6ETF) and *E*. *coli* MoeA (PDB 1G8L), structural superposition revealed discrete substitutions surrounding the oxyanion-binding site (Fig. 6*A*; Fig. S8). Docking analyses revealed a pronounced shift in metal preference, with MoeA1 binding WO_4_^2-^ with substantially higher affinity (−41.2 kcal mol^-1^) than MoO_4_^2-^ (−23.0 kcal mol^-1^), whereas canonical enzymes favored molybdate, consistent with their established specificity (37) (Fig. 6*B*).

In MoeA1, tungstate coordination is stabilized by an expanded hydrogen-bonding and electrostatic network involving Ile287, Lys288, Pro289, Gly290, Arg291, and Arg353; whereas the molybdate-bound complex forms fewer directional contacts, consistent with its reduced affinity (Fig. 6*C*; Fig. S9*A*). This reversal in metal preference is supported by MoeA1-specific substitutions that reshape the binding environment. Arg291 (corresponding to Lys297 in Cnx1E) directly engages WO_4_^2-^, whereas tungstate binding disrupts the Gly296-Lys297 configuration in Cnx1E, impairing optimal oxyanion positioning (37) (Fig. S9*B*). Similarly, the polar serine that orients MoO_4_^2-^ in Cnx1E (Ser328; Fig. 6*D*) and *E*. *coli* MoeA (Ser303; Fig. 6*E*) is replaced by Ala312 in MoeA1, disfavoring molybdate coordination.

To evaluate the functional contribution of these residues in metal discrimination, substitutions restoring canonical Mo-insertase features (R291K and A312S) were introduced and analyzed in the Δ*moeA1* background. WT MoeA1 supported efficient MeFDH1 maturation under W-supplemented conditions but showed minimal activity in molybdate-only medium (Fig. S10). In contrast, the R291K variant retained substantial W-dependent activity while also supporting MeFDH1 activation under molybdate-supplemented conditions, accompanied by detectable Mo incorporation (0.17 mol Mo per mol enzyme). The A312S substitution produced a similar but less pronounced effect, consistent with a supporting role in shaping the metal-binding environment. Because endogenous MoeA2 is retained in this background, the increased Mo incorporation cannot be attributed solely to the mutant MoeA1 variants and instead reflects the contribution of the MoeA1 pocket to metal discrimination in the native cellular context, rather than providing direct evidence for mutant MoeA1-mediated Mo insertion.

Metal selectivity under competitive conditions was further assessed using mixed-metal supplementation experiments in the Δ*moeA2* background expressing MeFDH1. Although MeFDH1 activity decreased under mixed-metal conditions relative to tungstate-only supplementation, inductively coupled plasma-optical emission spectrometry (ICP-OES) analysis showed that W remained the predominant incorporated metal, with Mo signals remaining low even under more than 30-fold molybdate excess (Fig. S11). These findings indicate that, despite competition at the level of the insertion pathway, MoeA1-mediated cofactor assembly remains strongly biased toward W.

Together, these data establish MoeA1 as a key determinant of W selectivity during cofactor assembly, providing a mechanistic basis for the dedicated W-dependent maturation pathway in *M*. *extorquens*.

## Discussion

This study defines a tungsten-specific pathway for MeFDH1 cofactor assembly in *M*. *extorquens* that diverges from the canonical molybdenum branch, orchestrated by MoeA1 and MobB as defining components that promote W-selective maturation, whereas MoeA2 supports the parallel Mo pathway. The division of labor establishes a mechanistic boundary between the two metal systems, ensuring precise delivery of W-bis-MGD to MeFDH1 without compromising Mo metabolism (38).

Comparable metal specialization has been documented in *Desulfovibrio* species, where distinct *moeA* paralogs are differentially expressed under W- or Mo-rich conditions, indicating adaptive segregation of the two maturation branches (17, 38, 39). This specialization is also supported by phylogenetic linkages to transporters, in which W-specific TupABC/WtpABC transporters are often collocated with W-utilization operons (17, 38, 40). Similarly, the *moeA1-mobB-tupBCA* gene cluster in *M*. *extorquens* suggests coordinated regulation of W uptake and cofactor assembly (Fig. S12), reinforcing the notion that W-specific transport and cofactor biogenesis operate as a linked functional unit in this organism.

The decisive divergence between Wco and Moco biosynthesis occurs at the step of metal ligation. In this reaction, members of the MoeA family coordinate and deliver either molybdate or tungstate to the dithiolene coordination sphere, thereby defining the identity of the finished cofactor (17, 41, 42, 43). In *M*. *extorquens*, MoeA1 performs this role specifically for MeFDH1 cofactor assembly, functioning as the dedicated tungstate insertase within the W-branch maturation pathway.

Docking and sequence analysis together indicate that *M*. *extorquens* MoeA1 has remodeled its active-site electrostatics to favor WO_4_^2-^ incorporation. Local enrichment of basic residues and loss of polar hydrogen-bond donors enhance tungstate stabilization while preserving the ancestral MPT-binding core. Functionally, this specialization aligns with cellular physiology: MoeA1 is strictly required for maturation of the W-dependent MeFDH1 but dispensable for Mo-dependent pathways.

Mutagenesis experiments provide functional support for this model. Substitution of Arg291 or Ala312, residues predicted to shape the electrostatic environment of the insertion pocket, reduced the efficiency of W-dependent MeFDH1 maturation while increasing Mo-dependent activity and Mo occupancy relative to WT MoeA1 under molybdate-supplemented conditions. Because these effects were observed in a genetic background that retains endogenous MoeA2, the data are most consistent with a role for the MoeA1 pocket in influencing metal selection during cofactor assembly *in vivo*, rather than demonstrating that the mutant MoeA1 variants alone mediate Mo insertion.

Under mixed-metal competition, when molybdate was supplied together with tungstate, W remained the predominant metal detected in purified MeFDH1 despite substantial molybdate excess, indicating that MoeA1-mediated cofactor assembly is not dictated solely by metal availability. The concurrent reduction in activity together with W occupancy suggests that molybdate can compete for interaction at or upstream of the insertion step but does not efficiently drive MeFDH1 maturation. These findings support a model in which the MoeA1 pocket enforces W-biased cofactor assembly by selectively stabilizing tungstate during insertion while limiting productive engagement of molybdate.

These features position *M*. *extorquens* MoeA1 between canonical Mo-insertases and specialized W-specific enzymes from anaerobes, suggesting an evolutionary trajectory toward W preference. Although a tungstate-bound MoeA1 structure is not yet available, the comparative analyses pinpoint residues that systematically diverge between W-biased and Mo-specific MoeAs, offering clear mechanistic hypotheses for how modest sequence changes reprogram metal specificity within this enzyme family.

Metal fidelity within the W branch of *M*. *extorquens* is reinforced by the recruitment of the accessory protein MobB. Our genetic analyses show that MobB is indispensable for W-dependent MeFDH1 maturation yet dispensable for the Mo-dependent pathway, identifying it as a defining component of the W-specific maturation branch. By contrast, in many Mo-centric bacteria such as *E*. *coli*, MobB is a nonessential, GTP-binding accessory that stabilizes MobA-dependent guanylylation machinery without dictating metal selectivity (25, 44). This functional divergence suggests that MobB has acquired a specialized role within the W pathway of *M*. *extorquens*.

The mechanistic basis for this branch-specific requirement remains to be fully resolved, but several possibilities emerge from our interaction and structural analyses. One possibility is that MobB helps organize or channel W-bound pterin intermediates (W-MPT) during cofactor assembly. Because W and Mo differ in coordination chemistry and redox behavior, W-MPT may require additional spatial organization to prevent mismetalation or premature oxidation. In this scenario, MobB could facilitate productive handling of the MoeA1-associated W-MPT prior to guanylylation. Alternatively, MobB may act as an allosteric regulator of the tungstate insertase. The robust MoeA1-MobB interaction detected by BACTH and AF3 analyses suggests that MobB binding could modulate the conformation or catalytic efficiency of MoeA1, thereby enhancing metal discrimination or promoting productive cofactor transfer. Notably, the *moeA1-mobB* gene synteny supports their functional coupling and suggests a coevolved insertase-scaffold module that enforces Wco fidelity. The presence of MoeA-MobB fusion proteins in several γ-proteobacteria further points to an evolutionarily conserved architectural solution for W-biased systems (28).

Downstream steps—from guanylylation to sulfuration and cofactor delivery—follow the established logic of Moco biosynthesis (24). MobA functions as the guanylyltransferase that attaches GMP to metal-bound MPT to generate the dinucleotide cofactor MGD (28, 29, 30, 31, 32, 45); whereas FdhD is a conserved FDH-dedicated chaperone-sulfurtransferase that binds bis-MGD, installs the terminal sulfido ligand, and loads the matured cofactor onto the apoenzyme (46, 47, 48, 49, 50). These biochemical roles integrate seamlessly with our proposed model: MobA executes downstream of the MoeA1-MobB complex, mediating guanylylation and subsequently engages FdhD to complete sulfuration and cofactor delivery.

In contrast to the canonical Moco pathway in *E*. *coli*, where MoeA, MogA, and MobA operate within a coordinated cytosolic module to generate and insert Mo-bis-MGD (33, 34, 51), the *Methylorubrum* system adopts a more distributed and W-selective architecture. Rather than invoking a permanently assembled multienzyme complex, the late-stage machinery is best interpreted as a modular relay in which distinct interactions support ordered cofactor processing and transfer. This organization may enhance metal specificity and kinetic efficiency without requiring a preassembled megacomplex. Mechanistically, such organization could help maintain cofactor shielding, limit mismetalation, and streamline the final transfer of W-bis-MGD to MeFDH1—the terminal FDH.

The MoeA1-centered relay proposed here thus represents a functional refinement of ancestral Moco biosynthesis—retaining the chemical logic of adenylylation, metal insertion, and guanylylation while executing these reactions through successive interactions that enhance cofactor fidelity and turnover efficiency. These results underscore how aerobic W enzymes have reconfigured conserved Moco biosynthetic components into a flexible, sequential network that preserves catalytic precision while accommodating the redox reactivity of W.

## Conclusion

The discovery of a MoeA1-centered tungsten maturation branch in *M*. *extorquens* provides a mechanistic framework for understanding how closely related oxyanions are selectively routed into distinct metalloenzymes within a single aerobic organism. By delineating the genetic architecture of this W-specific pathway and identifying residue-level determinants that tune metal discrimination, this study extends current models of cofactor biosynthesis beyond pathway segregation to include insertase-level selectivity.

Beyond elucidating the mechanism of enzyme maturation, this work provides a molecular foundation for designing synthetic maturation systems capable of supporting W enzymes in industrial hosts such as *E*. *coli*. More broadly, it illustrates how conserved cofactor biosynthetic machinery can be repurposed through modular specialization to enforce metal fidelity, opening opportunities for bioengineering robust CO_2_-reducing biocatalysts that exploit the superior redox properties of Wco under aerobic or electrochemical conditions.

## Experimental procedures

### Comparative genomics and identification of Moco/Wco biosynthetic proteins

Comparative genomic analyses were performed to identify homologs of Moco/Wco biosynthetic proteins in *M*. *extorquens*. Protein sequences for established *E*. *coli* Moco pathway components were retrieved from UniProtKB and used as queries in BLASTP searches against the *M*. *extorquens* proteome using default parameters. Percent sequence identities were obtained directly from BLAST alignments, and UniProt accessions and *M*. *extorquens* locus identifiers for all identified homologs are presented in Table 1.

### Construction of *M*. *extorquens* gene deletion strains

Deletions of target loci were generated by Cre-*loxP* allelic exchange as previously described (52). Briefly, approximately 0.5 to 0.6 kb regions immediately upstream and downstream of each open reading frame were PCR-amplified with FailSafe PCR Enzyme Mix (Lucigen) and inserted into the allelic-exchange vector pCM184 by SLIC (53). The resulting constructs were introduced into *M*. *extorquens* by electroporation, and integrants were selected on tetracycline- and kanamycin-containing plates. Following allelic exchange, candidate deletions were verified by colony PCR and confirmed by sequencing. A complete list of strains and primers is provided in Tables S3 and S4.

### Plasmid construction and homologous expression of MeFDH1

The MeFDH1 expression plasmid pCM110-MeFDH1 was constructed as described by Jang *et al*. (13). The *fdh1A* (α) and *fdh1B* (β) genes were PCR-amplified from *M*. *extorquens* genomic DNA and assembled into the broad-host-range pCM110 under the *P*_*mxaF*_ promoter. A C-terminal His_6_ tag was fused to *fdh1a* to enable purification by Ni^2+^-affinity chromatography.

For genetic complementation, MeFDH1 was cloned into the multiple cloning site 1 of pCM110Duet (tetracycline selectable), and candidate Wco biosynthetic genes were inserted into multiple cloning site 2 by sequence- and ligation-independent cloning. All plasmids were sequence-verified and introduced into the indicated deletion strains by electroporation, with transformants selected on tetracycline-containing medium. Plasmids and inserts used in this study are listed in Table S3.

### Strains, media, and cultivation

*M*. *extorquens* derivatives (WT and deletion strains) were cultivated in mineral salts medium as described previously, using succinate as the carbon source and 30 μM Na_2_WO_4_ to support MeFDH1 expression (13). The base medium contained (per liter): 1.62 g NH_4_Cl, 0.20 g MgSO_4_, 2.21 g K_2_HPO_4_, and 1.25 g NaH_2_PO_4_·2H_2_O, supplemented with a trace-element solution comprising 15 mg Na_2_EDTA·2H_2_O, 4.5 mg ZnSO_4_·7H_2_O, 0.30 mg CoCl_2_·6H_2_O, 1.0 mg MnCl_2_·4H_2_O, 1.0 mg H_3_BO_3_, 2.5 mg CaCl_2_, 0.40 mg Na_2_MoO_4_·2H_2_O, 3.0 mg FeSO_4_·7H_2_O, and 0.30 mg CuSO_4_·5H_2_O. For metal-selectivity experiments, the basal trace-element solution was used without modification. Accordingly, tungstate-supplemented conditions refer to medium containing 30 μM Na_2_WO_4_ with no additional molybdate beyond the basal trace-metal mix.

For molybdate-grown conditions, cultures received 30 μM Na_2_MoO_4_ with no added tungstate. Cultures were incubated at 30 °C with shaking (200 rpm) in baffled flasks at a 1:5 culture-to-flask volume ratio and growth was monitored by optical density at 600 nm (OD_600_) on a UV-Vis spectrophotometer (Shimadzu). At mid-exponential phase (OD_600_≈ 0.6–0.8), methanol was added to a final concentration of 0.5% (v/v) to induce recombinant expression. After 48 h, cells were harvested by centrifugation (7000 rpm, 15 min, 4 °C) and pellets were stored at −70 °C until use. Where relevant, antibiotics for *M*. *extorquens* were included at: kanamycin, 50 μg mL^-1^; rifamycin, 50 μg mL^-1^; tetracycline, 10 μg mL^-1^.

### SDS-PAGE and immunoblotting

Cell pellets harvested at mid-exponential phase were resuspended in buffer A (50 mM MOPS/KOH, pH 7.0, 200 mM NaCl, 20 mM imidazole), disrupted by sonication on ice, and clarified by centrifugation. Protein concentrations were determined using the bicinchoninic acid (BCA) assay. For SDS-PAGE, 20 μg of total protein was mixed with Laemmli sample buffer, heated at 95 °C for 5 min, and separated on 12% Mini-PROTEAN TGX precast gels (Bio-Rad) at 200 V for 40 min in Tris-glycine running buffer. Proteins were transferred to polyvinylidene difluoride membranes using the Trans-Blot Turbo system (Bio-Rad). Immunodetection was performed with the iBind Flex Western Device (Thermo Fisher Scientific) according to the manufacturer’s protocol. Primary antibodies against His-tag (Abcam) and MeFDH1 β-subunit (ABFRONTIER) were used at 1:1000 dilutions, followed by alkaline phosphatase-conjugated secondary antibodies at 1:2000 dilutions. Detection was carried out using BCIP/NBT substrate solution (Sigma-Aldrich), and blot images were captured using a Gel-Doc XR imaging system (Bio-Rad).

### Protein purification

MeFDH1 produced in tungstate-grown *M*. *extorquens* was purified by Ni^2+^-affinity chromatography on Ni-NTA affinity resin (Qiagen). Cell pellets were resuspended at 10 ml g^-1^ wet weight in buffer A (50 mM MOPS/KOH, pH 7.0, 200 mM NaCl, 20 mM imidazole) and disrupted by sonication on ice (40% amplitude, 2 s on/2 s off). Cell debris was removed by centrifugation (11,000 rpm, 30 min, 4 °C). The clarified supernatant was loaded onto Ni-NTA pre-equilibrated in buffer A, washed with five column volumes of buffer A, and eluted with buffer B (buffer A supplemented with 300 mM imidazole). Protein concentration was determined by absorbance at 280 nm using a NanoDrop spectrophotometer (Thermo Fisher Scientific) and a mass extinction coefficient calculated with EMBOSS pepstats (ε = 8.94 L mg^-1^cm^-1^).

### Enzyme activity assays

CO_2_-reducing activity of purified MeFDH1 was measured spectrophotometrically using reduced ethyl viologen (EV) as the artificial electron donor. Assays were performed anaerobically at 30 °C in sealed cuvettes containing assay buffer (200 mM potassium phosphate, pH 6.5) supplemented with 100 mM sodium bicarbonate as substrate. EV was reduced with excess zinc metal immediately prior to use. Reactions were initiated by addition of enzyme, and CO_2_ reduction was monitored by the decrease in absorbance of reduced EV at 600 nm (ε_600_ = 10.22 mM^-1^cm^-1^). Initial rates were calculated from the linear portion of the traces and normalized to enzyme concentration.

Formate dehydrogenase activity in whole-cell extracts from molybdate-grown cultures was determined by monitoring NAD^+^-dependent formate oxidation. Cells were harvested in mid-exponential phase, washed, and disrupted by sonication under aerobic conditions. Clarified lysates were assayed at 30 °C in buffer containing 50 mM MOPS/KOH (pH 7.0), 30 mM sodium formate, and 0.5 mM NAD^+^. Reactions were initiated by addition of lysate, and NADH formation was followed at 340 nm (ε_340_ = 6.22 mM^-1^cm^-1^). Activities were calculated from initial linear rates and normalized to total protein content.

### Metal quantification by inductively coupled plasma-optical emission spectrometry

Elemental analysis of purified MeFDH1 samples was performed by ICP-OES. Protein samples (2–5 mg) were digested in 12 ml of trace-metal-grade HNO_3_ and 1 ml of HF at 200 °C until clear, then diluted to a final volume of 50 ml with 1% (v/v) HNO_3_. Measurements were conducted using a Varian 720 ES using PerkinElmer Multi-Element Calibration Standards 3 and 5 for external calibration. Instrumental detection limits were 0.016 mg kg^-1^ (Mo) and 0.025 mg kg^-1^(W). Metal contents (mol metal mol^-1^ enzyme) were calculated from measured concentrations and protein molarity.

### BACTH protein-protein interaction assays

Protein-protein interactions were assessed using the Euromedex BACTH system with minor modifications. Coding sequences for MoeA1, MobA, MobB, MoaB, FdhD, and the MeFDH1 α- and β-subunits were PCR-amplified and cloned into pKT25 and pUT18 C to generate N- or C-terminal fusions to the T25 or T18 fragments. All constructs were sequence-verified. Reciprocal plasmid pairs were co-transformed into *E*. *coli* BTH101 and plated on LB agar containing ampicillin (100 μg mL^-1^), kanamycin (50 μg mL^-1^), IPTG (0.5 mM), and X-gal (40 μg mL^-1^). After incubation at 30 °C for 24–48 h, colonies were scored qualitatively by color. For quantitative analysis, *E*. *coli* BTH101 cultures were grown to mid-log phase at 30 °C in LB supplemented with antibiotics and IPTG (0.5 mM). β-galactosidase activity was determined using the Promega β-Galactosidase Enzyme Assay System with Reporter Lysis Buffer, according to the manufacturer’s instructions. Activities are reported as Miller units calculated as described in Olson *et al*. (54). The strains, plasmid pairs, and fusion orientations used for BACTH are detailed in Table S5.

### AlphaFold 3 structure prediction

Protein structures of *M*. *extorquens* MoeA1, MobA, MobB, FdhD, and protein complexes were predicted using AlphaFold 3 with the default multimer settings (35). Model quality was evaluated using predicted local distance difference test (pLDDT) scores, and complex confidence was assessed using the ipTM + pTM composite score, which serves as the ranking metric for multimer predictions as described by Evans *et al*. (55). For each target, the top-ranked model was retained and inspected in UCSF ChimeraX and BIOVIA Discovery Studio Visualizer. predicted aligned error viewer was used for the interactive visualization of the predicted aligned error for multimer structure predictions.

### Multiple sequence alignment

Protein sequences were retrieved from the UniProt database, including both canonical Mo-dependent enzymes and W-associated homologs (accession numbers listed in Table S6). Multiple sequence alignments were generated using Clustal Omega with default parameters. Alignments were visualized and annotated in Jalview and manually curated to ensure accurate positioning of conserved motifs and metal-cofactor-associated residues. Consensus sequences were derived using a majority-rule approach across aligned positions to highlight conserved features distinguishing Mo- and W-associated lineages.

### Molecular docking simulations

Docking simulations were performed using the CDOCKER algorithm in BIOVIA Discovery Studio. Protein models were prepared by adding polar hydrogens, assigning CHARMm charges, and removing water molecules. Ligand structures for molybdate (MoO_4_^2-^) and tungstate (WO_4_^2-^) were obtained from PubChem and prepared similarly. Active-site regions were defined based on conserved residues corresponding to the cofactor-binding pockets in MoeA (PDB 1G8L) and Cnx1E (PDB 6ETF), and docking grids were centered over the metal-binding cleft. Simulations were run using default CDOCKER parameters, and resulting poses were ranked by binding energy (kcal mol^-1^). Protein-ligand interactions were analyzed in Discovery Studio Visualizer and UCSF ChimeraX.

### Site-directed mutagenesis of MoeA1

Site-directed mutagenesis was performed to evaluate the contribution of predicted metal-binding residues to tungsten selectivity. Substitutions R291K and A312S were introduced into the *moeA1* gene using PCR-based mutagenesis with mutagenic primers (Table S4). Mutant constructs were sequence verified and cloned into the pCM110-MeFDH1-MoeA1 plasmid. The resulting plasmids were introduced into the Δ*moeA1* strain expressing MeFDH1 by electroporation. Recombinant strains were cultivated under the same conditions used for WT complementation, and purified MeFDH1 was analyzed for enzymatic activity and metal content.

### Mixed-metal competition experiments

To assess metal selectivity during cofactor assembly, Δ*moeA2* strains expressing MeFDH1 were cultivated under tungstate-supplemented conditions described above or with additional molybdate at defined ratios (30 μM Mo: 30 μM W or 1000 μM Mo: 30 μM W). Cells were grown and induced under the same conditions used for enzyme production. MeFDH1 was purified by Ni^2+^-affinity chromatography, and enzyme activity and metal content were determined using the activity assays and ICP-OES procedures.

### Statistical analysis

All experiments were performed with at least *n* = 3 independent biological replicates, as indicated in the figure legends. Data are presented as mean ± SD. Statistical analyses were conducted using OriginPro (https://www.originlab.com). Comparisons between two groups were performed using two-tailed Student’s *t*-tests. Differences were considered statistically significant at *p* < 0.05.

## Data availability

All data supporting the findings of this study are available within the article and its Supporting Information.

## Supporting information

This article contains Supporting Information (56, 57, 58, 59, 60, 61, 62, 63, 64, 65, 66, 67, 68).

## Conflict of interest

The authors declare that they have no conflicts of interest with the contents of this article.
