# Supplementary material for: A tungsten-specific maturation pathway governs cofactor assembly of a CO2-reducing formate dehydrogenase in Methylorubrum extorquens[image]
Source: J Biol Chem. 2026 Apr 22;302(6):111483. doi: 10.1016/j.jbc.2026.111483 (PMC13197769; doi:10.1016/j.jbc.2026.111483)
Supplement: Supplementary Material 1 [file mmc1.docx]

Supporting Information

**A tungsten-specific maturation pathway governs cofactor assembly of a CO_2_-reducing formate dehydrogenase in *Methylorubrum extorquens***

*Running title: Tungsten-specific cofactor maturation in M. extorquens*

Uyen Thu Phan^1^, Yong Hwan Kim^1, 2,^ *

1. School of Energy and Chemical Engineering, Ulsan National Institute of Science and Technology, Ulsan 44919, Republic of Korea
2. Graduate School of Carbon Neutrality, Ulsan National Institute of Science and Technology, Ulsan 44919, Republic of Korea

- Corresponding author: Yong Hwan Kim ([metalkim@unist.ac.kr](mailto:metalkim@unist.ac.kr))

**This PDF file includes:**

Figures S1-S12

Tables S1-S6


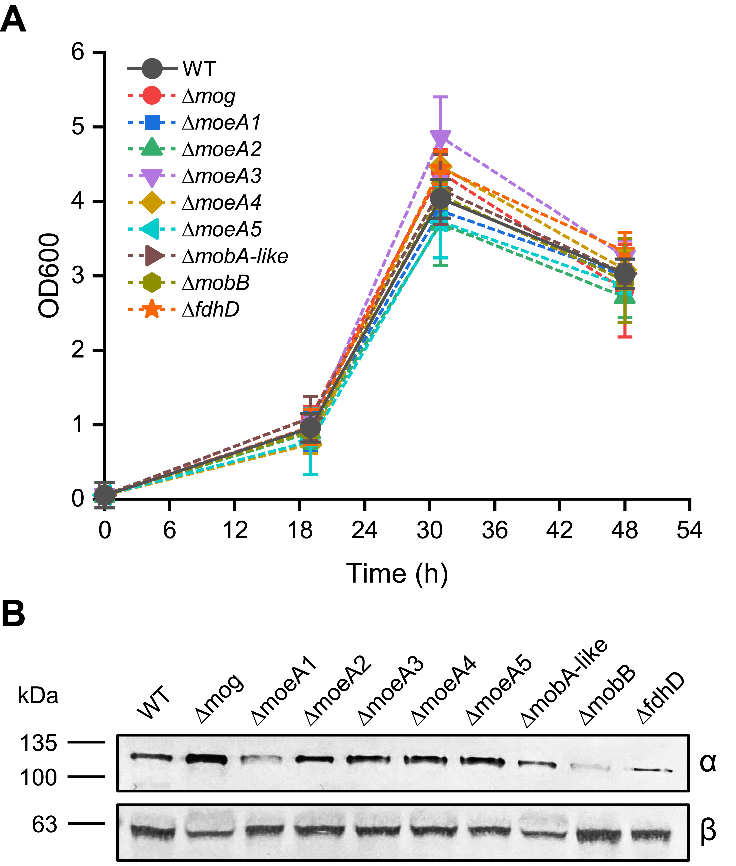


# Figure S1. Growth and expression of MeFDH1 in *Methylorubrum extorquens* deletion mutants.

**(A)** Growth curves of wild-type (WT) and deletion mutants expressing MeFDH1. Optical density at 600 nm (OD_600_) values were recorded over 48 h (mean ± SD, n = 3).

**(B)** Immunoblot analysis of MeFDH1 subunits in whole-cell lysates.


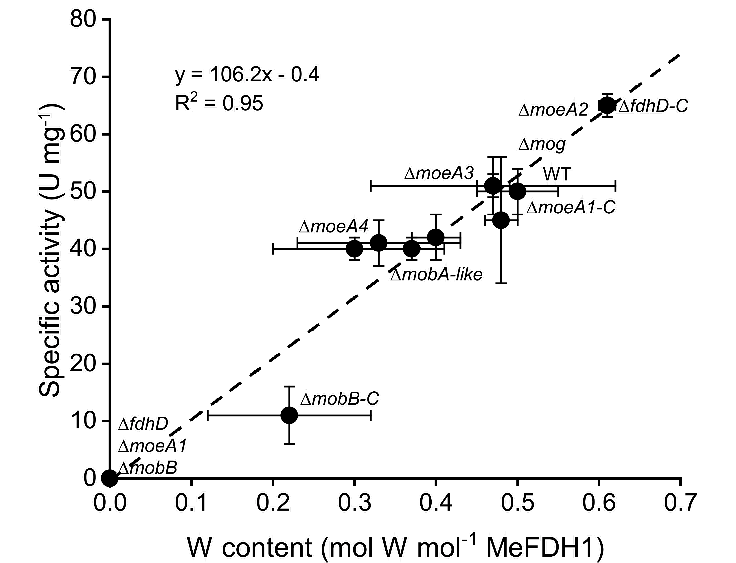


# Figure S2. Correlation between tungsten incorporation and MeFDH1 activity under tungstate supplementation.

Inductively coupled plasma optical emission spectrometry (ICP-OES) analysis of purified MeFDH1 from tungstate-supplemented *Methylorubrum extorquens* strains demonstrated a strong linear relationship between tungsten content and specific activity (R^2^ = 0.95), confirming its strict tungsten dependence.


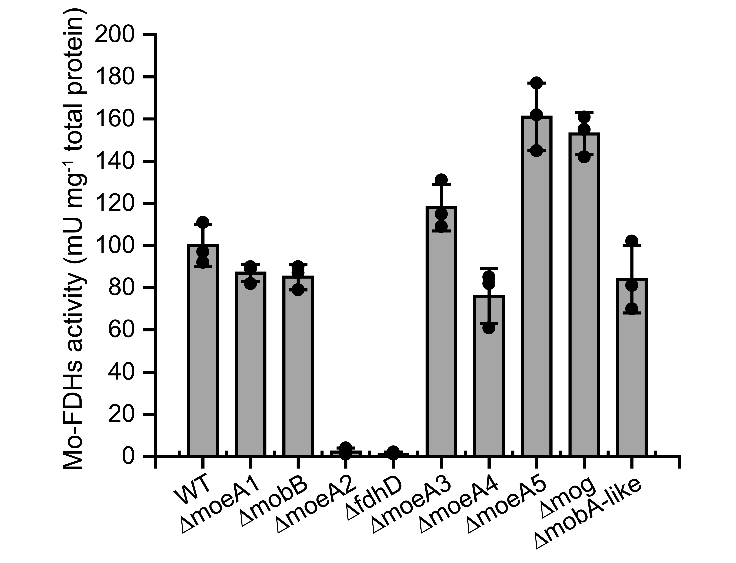


# Figure S3. Activity of endogenous Mo-dependent FDHs in *Methylorubrum extorquens* strains lacking MeFDH1 grown with molybdate.

Wild-type (WT) and deletion strains were cultivated in the presence of molybdate and assayed in clarified cell-free extracts using NAD^+^ as the electron acceptor. Activities are reported as mU mg^-1^ total protein. Bars represent mean ± SD (n = 3). Because MeFDH1 is absent in all strains, the measured activities exclusively reflect Mo-dependent FDHs.


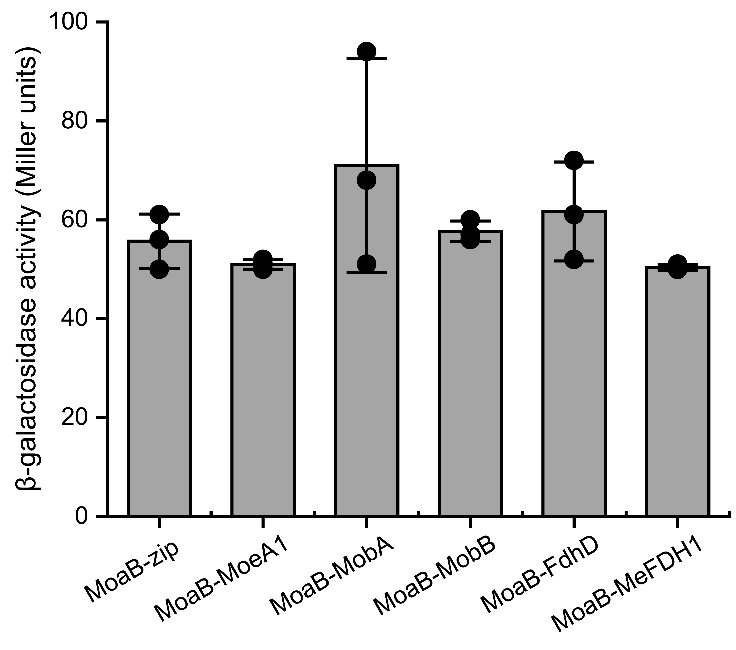


# Figure S4. BACTH analysis of MoaB interactions

Pairwise BACTH assays involving MoaB were performed and quantified as described in Fig. 3. All MoaB-containing combinations yielded β-galactosidase activity indistinguishable from the zip negative-control baseline.

**
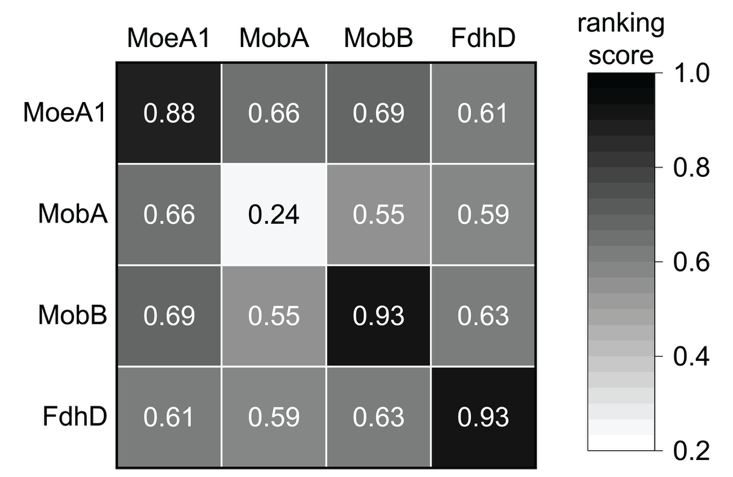
**

# ****Figure S5. Pairwise AlphaFold 3 ranking score heat map for Wco pathway components.****

Heat map summarizing AlphaFold 3 (AF3) ranking scores for all pairwise combinations tested among Wco biosynthesis and delivery proteins. Scores reflect overall interface confidence derived from AF3 multimer predictions, incorporating ipTM and pTM metrics used for model ranking.


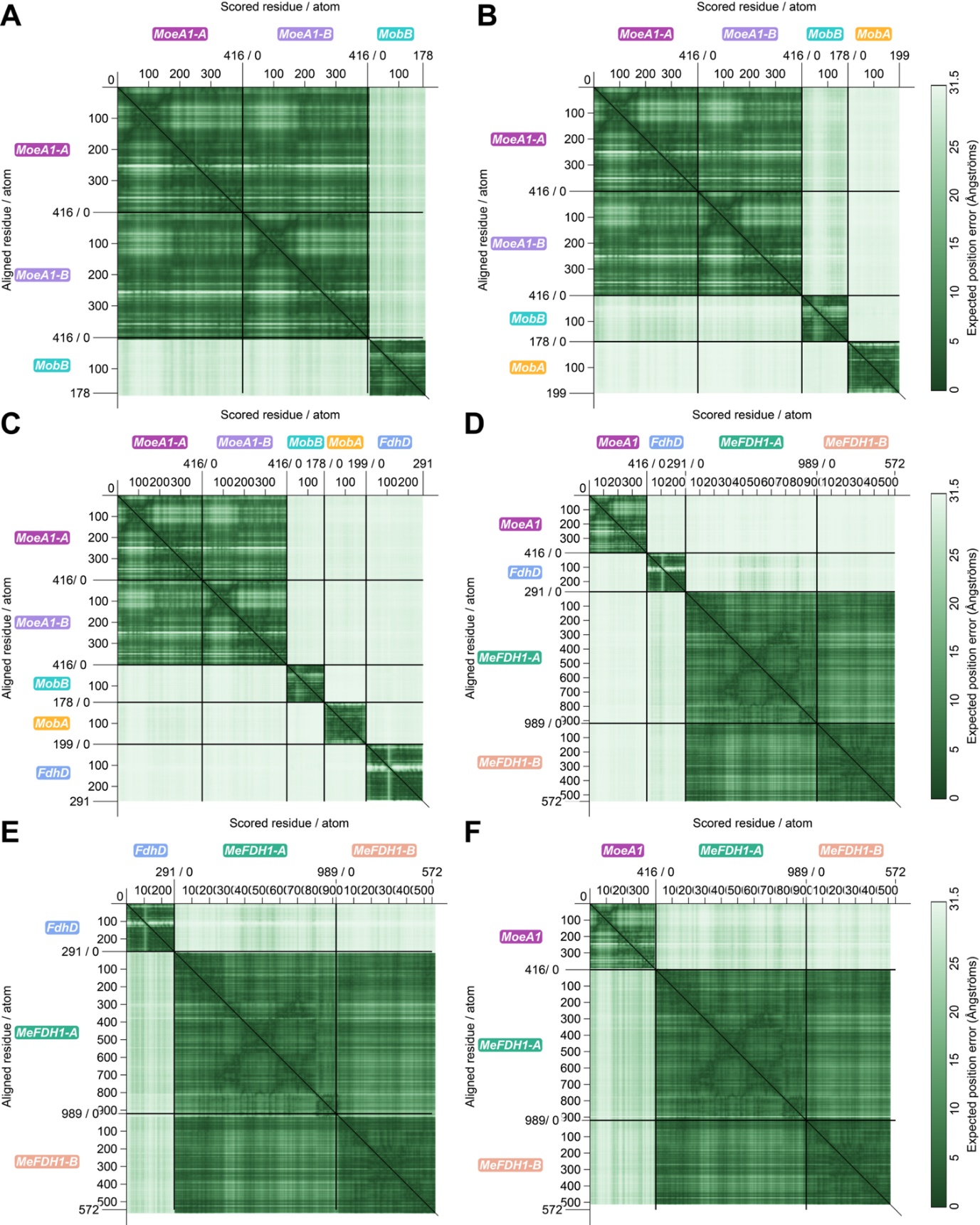


# ****Figure S6. Predicted aligned error (PAE) maps for AF3-modeled protein pairs.****

**(A)** MoeA1-MobB complex showing a well-localized low-PAE interface between MoeA1 and MobB, consistent with the high ipTM score and strong BACTH signal.

**(B)** MoeA1-MobB-MobA ternary model exhibiting diffuse inter-subunit PAE despite well-resolved monomer folds, suggesting a less well-defined association.

**(C)** MoeA1-MobB-MobA-FdhD higher-order assembly displaying broadly elevated inter-chain PAE, consistent with low-confidence higher-order arrangements.

**(D)** MoeA1-FdhD-MeFDH1 model in which FdhD engages the MeFDH1 α-subunit with moderate interface confidence, compatible with a delivery role during sulfuration-coupled cofactor transfer.

**(E)** FdhD-MeFDH1 complex showing localized low-PAE contacts at the delivery interface.

**(F)** MoeA1-MeFDH1 model positioning MoeA1 adjacent to the apoenzyme surface with moderate interface confidence.


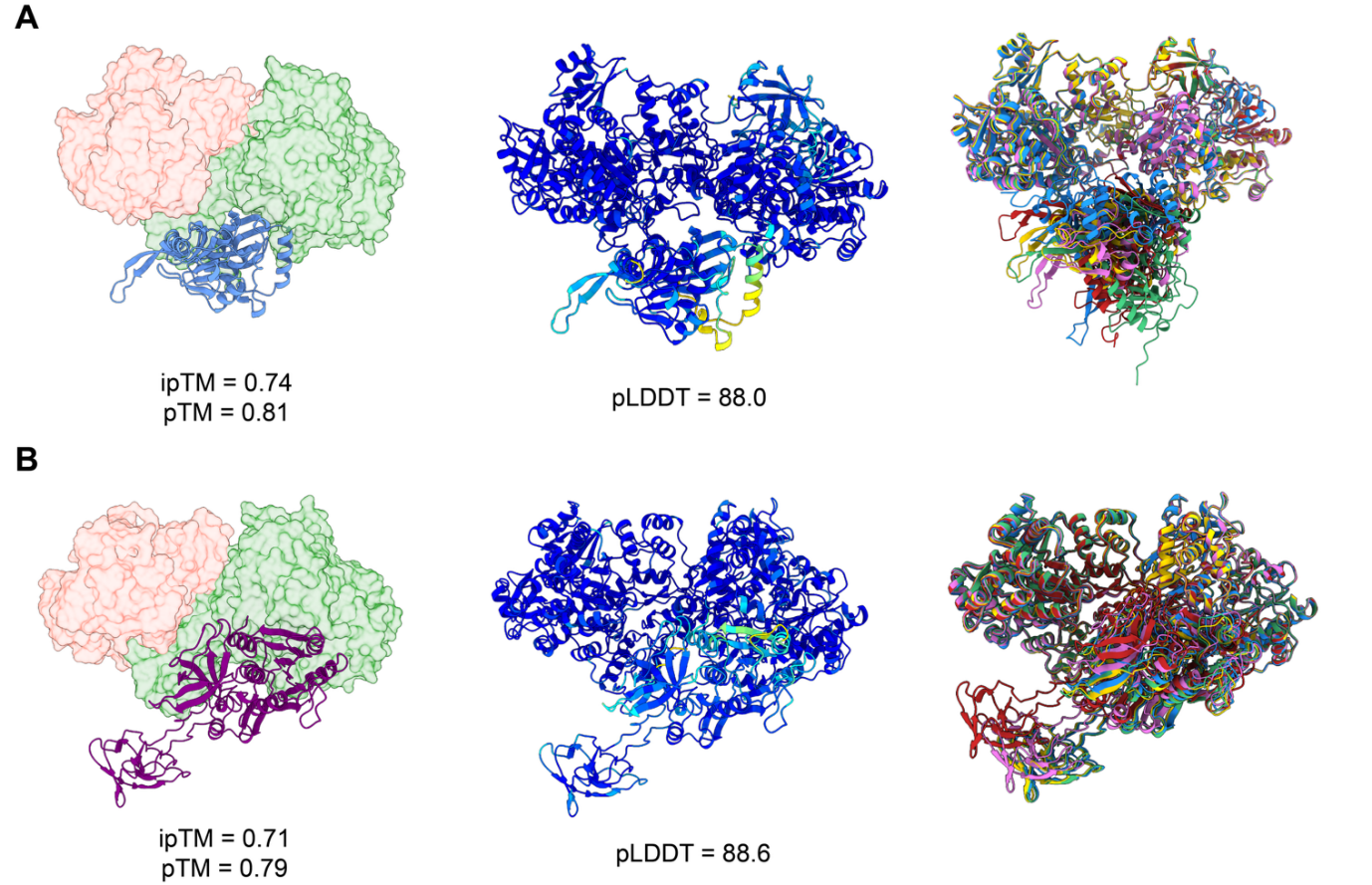


# Figure S7. AlphaFold 3 (AF3) models of MeFDH1 delivery and insertase engagement.

Left panels show the overall assemblies with predicted confidence metrics (ipTM and pTM). Middle panels display the AF3 rank-1 models colored by pLDDT, and right panels show overlays of the top five AF3 models.

**(A)** AF3-predicted FdhD-MeFDH1 complex, illustrating docking of FdhD (blue) onto the MeFDH1 α-subunit (green).

**(B)** AF3-predicted MoeA1-MeFDH1 complex, positioning MoeA1 (purple) adjacent to the apoenzyme interface, providing a structural rationale for MoeA1 specificity in MeFDH1 activation and cofactor delivery.

**
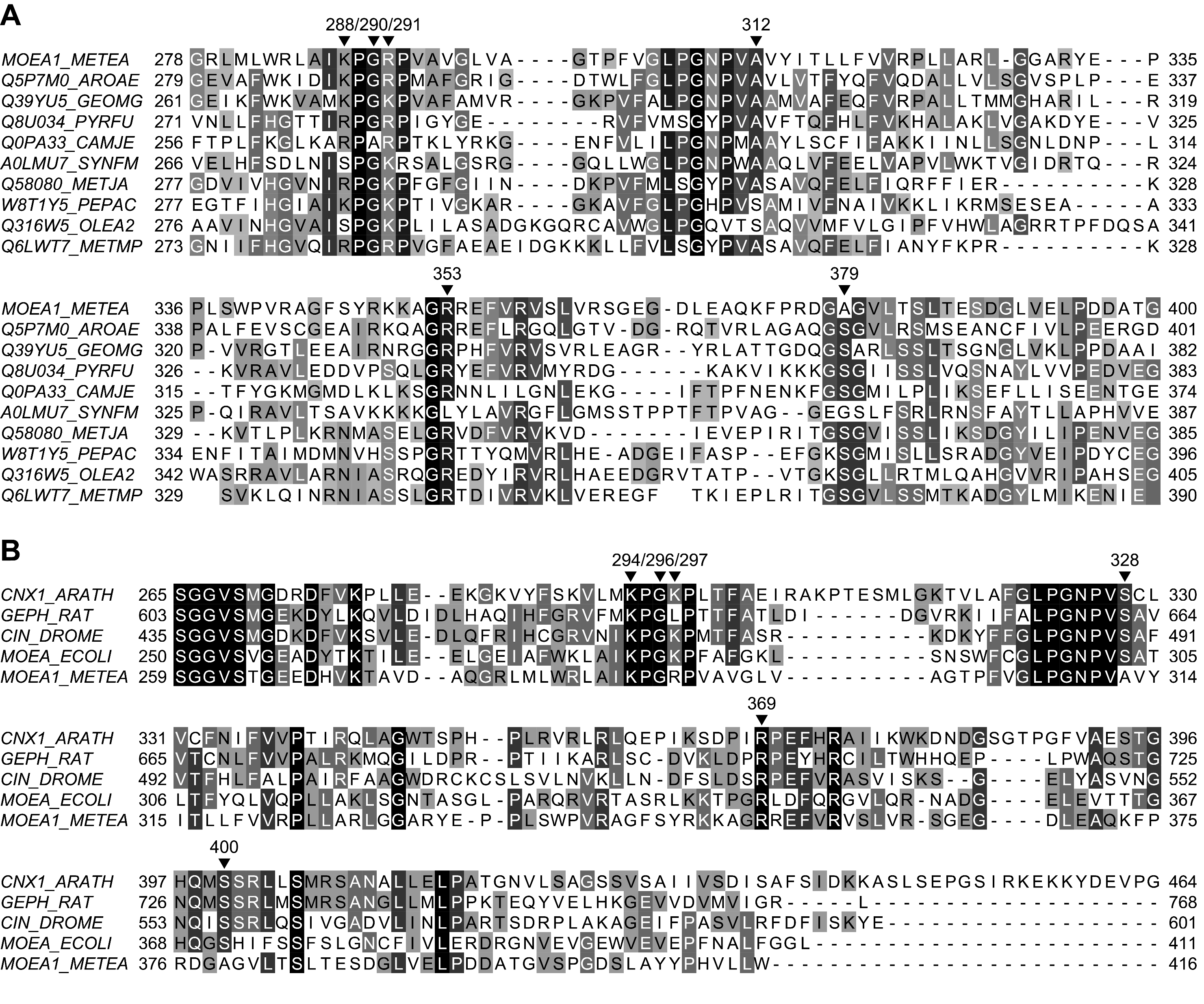
**

# Figure S8. Sequence features distinguishing W-selective MoeA1 homologs from canonical Mo-insertases.

**(A)** Multiple sequence alignment of representative putative W-specific MoeA homologs generated using Clustal Omega. Sequences include *Methylorubrum extorquens* MoeA1 (MOEA1_METEA), *Aromatoleum aromaticum* (Q5P7M0_AROAE), *Geobacter metallireducens* (Q39YU5_GEOM6), *Pyrococcus furiosus* (Q8U034_PYRFU), *Campylobacter jejuni* (Q0PA33_CAMJE), *Syntrophobacter fumaroxidans* (A0LMU7_SYNGM), *Methanocaldococcus jannaschii* (O58080_METJA), *Eubacterium acidaminophilum* (W8T1V5_PEPAC), *Desulfovibrio alaskensis* (Q316W5_OLEA2), and *Methanococcus maripaludis* (Q6LWT7_METMP). Conserved features around the oxyanion-binding pocket are shaded, with MoeA1 numbering used for callouts.

**(B)** Alignment of canonical Mo-insertases – Cnx1E (*Arabidopsis thaliana*), GephE (*Rattus norvegicus*), CIN (*Drosophila melanogaster*), *E. coli* MoeA – together with *M. extorquens* MoeA1. Residues contributing to MoO_4_^2-^ positioning are shaded, with Cnx1E numbering used for interpretive callouts.


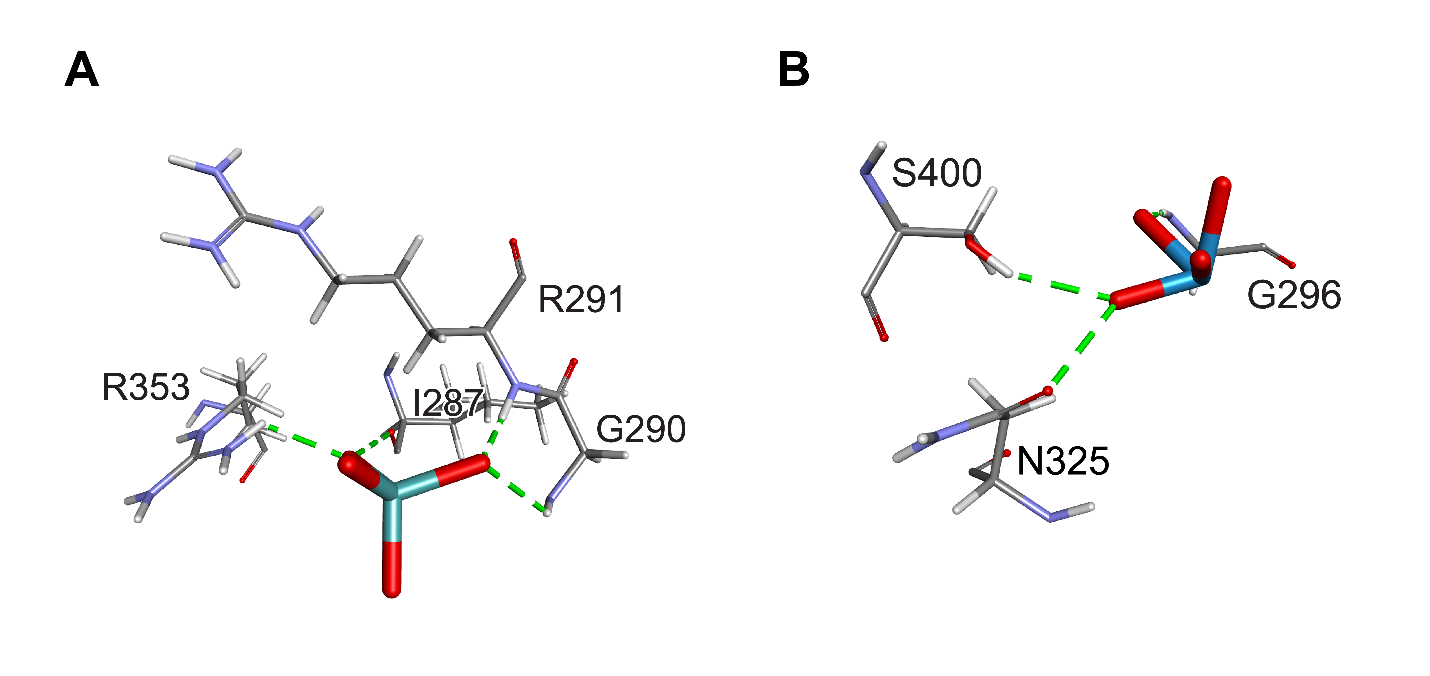


# Figure S9. Differential coordination of tungstate and molybdate in MoeA1 and Cnx1E.

**(A)** Docking model of molybdate bound to MoeA1, showing reduced and poorly oriented interactions relative to tungstate.

**(B)** Tungstate docked into the Cnx1E active site, illustrating disruption of the Gly296-Lys297 configuration and impaired oxyanion positioning compared with the native molybdate-binding mode.


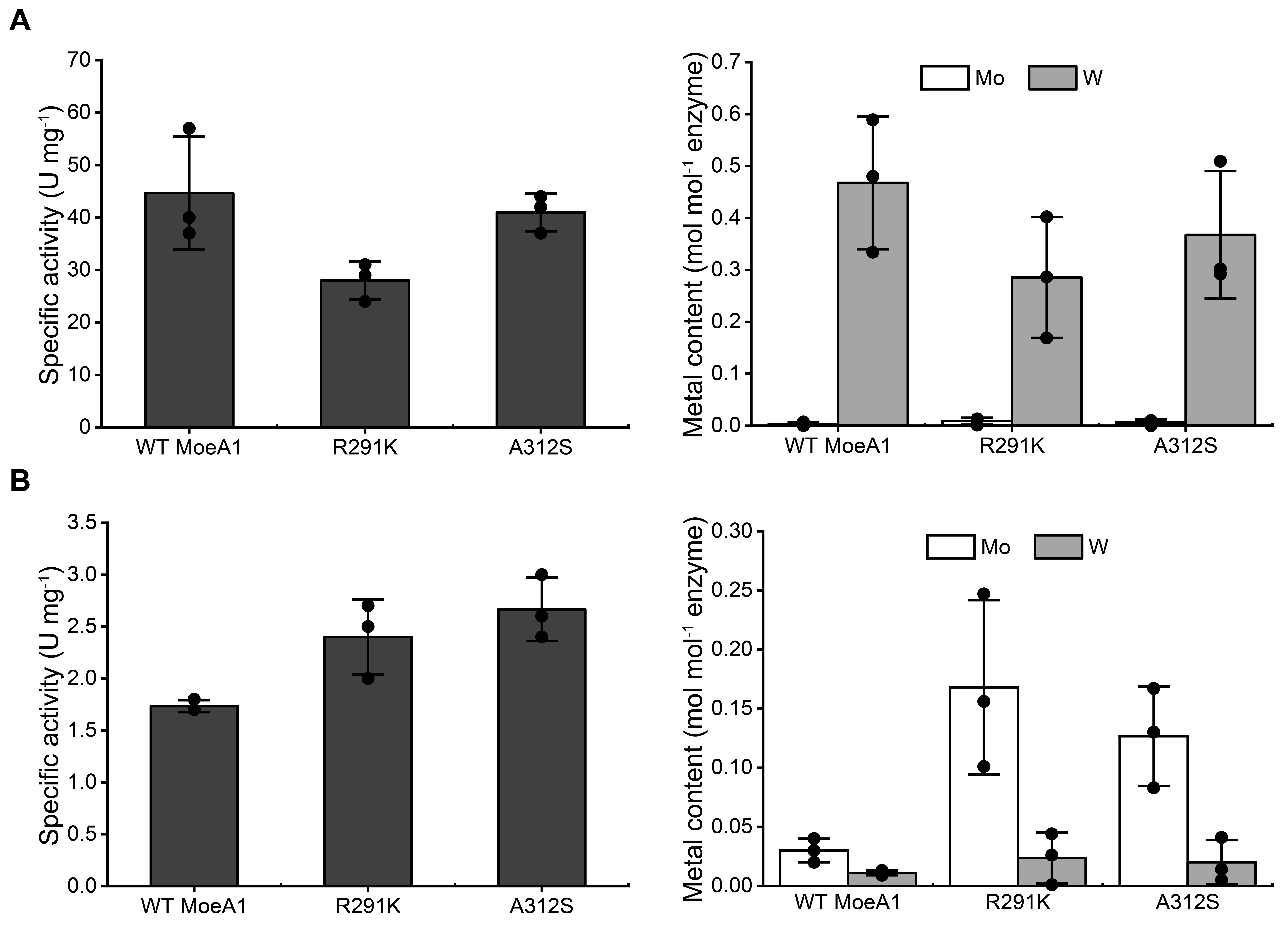


# Figure S10. Effects of MoeA1 variants on metal incorporation and enzyme activity.

Variants were expressed in the Δ*moeA1* background producing MeFDH1 and analyzed under defined metal supplementation conditions. Because MoeA2 is retained in this background, the observed Mo incorporation cannot be attributed solely to the mutant MoeA1 variants and instead reflects the contribution of the MoeA1 pocket to metal discrimination in the native cellular context.

**(A)** Specific activity (left) and metal content (right) of MeFDH1 produced with WT MoeA1, R291K, or A312S under tungsten conditions. W (gray) and Mo (white) contents are shown as mol metal per mol enzyme.

**(B)** Specific activity (left) and metal content (right) under molybdenum conditions.

Bars show mean ± SD (n = 3 biological replicates).


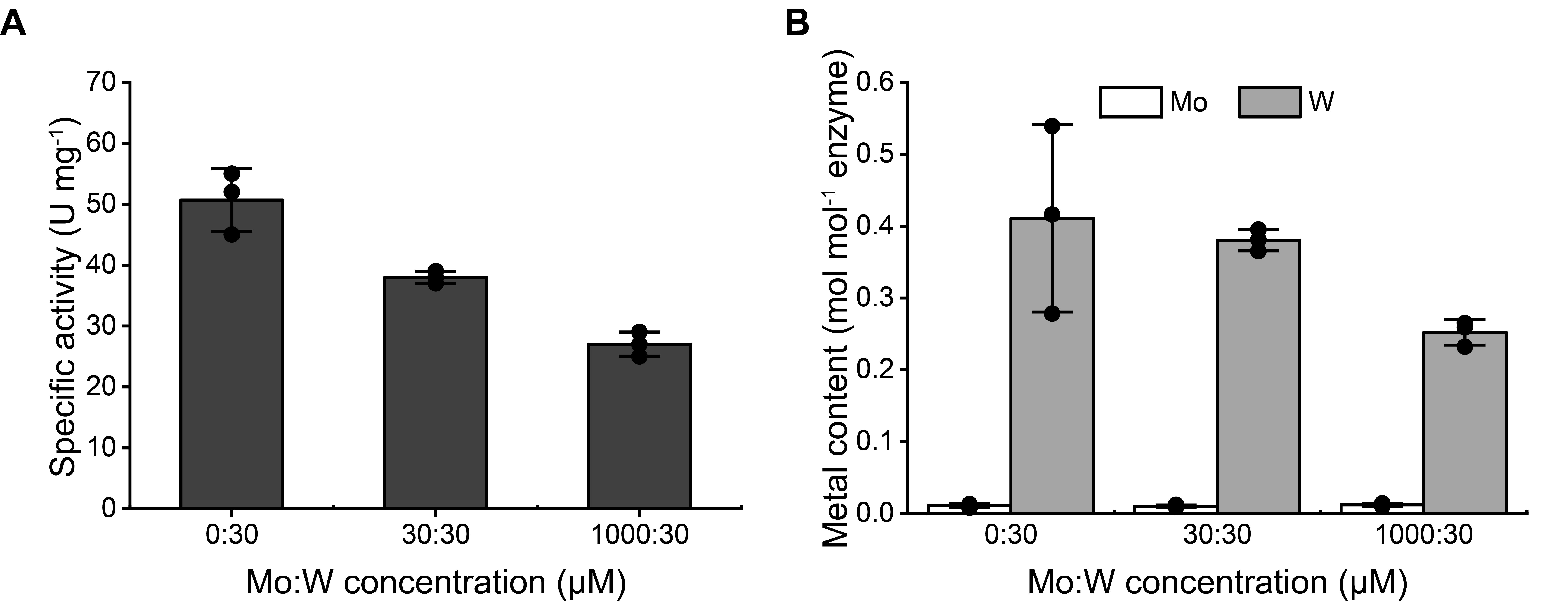


# Figure S11. Effect of Mo:W ratios on MeFDH1 activity and metal incorporation.

Experiments were performed in the Δ*moeA2* background to isolate the insertase-level contribution of MoeA1, ensuring that metal incorporation into MeFDH1 reflects MoeA1-dependent activity.

**(A)** Specific activity of MeFDH1 produced at varying Mo:W ratios (0:30, 30:30, 1000:30 µM).

**(B)** Metal content of purified enzyme showing W (gray) and Mo (white) as mol metal per mol enzyme.

Bars represent mean ± SD (n = 3 biological replicates).


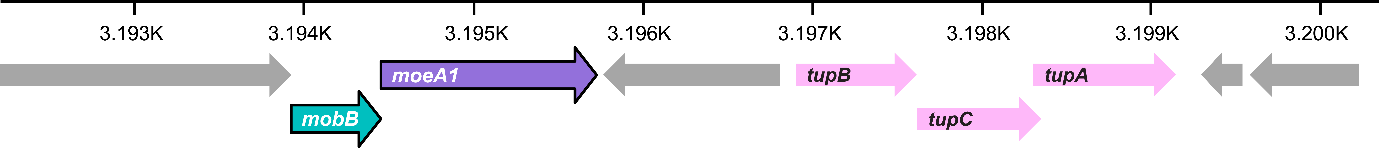


# ****Figure S12.**** Genomic organization of the *moeA1* locus in *Methylorubrum extorquens*.

Schematic representation of the chromosomal locus encompassing moeA1, mobB, and the tungsten transporter genes tupB, tupC, and tupA. Gene orientations are indicated by arrows, and genomic coordinates (kb) are shown above.

# Table S1. MeFDH1 activity and metal content of purified MeFDH1 from complemented *Methylorubrum extorquens* **mutants.**

MeFDH1 purified from wild-type (WT) and genetically complemented strains was assayed for CO_2_-reducing activity using reduced ethyl viologen (EV) as an artificial electron donor. Specific activities are expressed as units per milligram of MeFDH1 (U mg^-1^; mean ± SD, n = 3). Tungsten and molybdenum contents were quantified by ICP–OES and normalized to the molar concentration of MeFDH1. ND indicates values below the limit of detection.

| Strain | MeFDH1 specific activity (U mg^-1^) | Mo content (mol Mo mol^-1^ MeFDH1) | W content (mol W mol^-1^ MeFDH1) |
| --- | --- | --- | --- |
| WT | 50 **±** 4 | ND | 0.48 ± 0.03 |
| Δ*moeA1*-C | 45 **±** 11 | ND | 0.47 ± 0.13 |
| Δ*mobB*-C | 11 **±** 5 | ND | 0.23 ± 0.10 |
| Δ*fdhD*-C | 65 **±** 2 | ND | 0.61 ± 0.02 |

# Table S2. AlphaFold 3 multimer modeling statistics for multichain complexes among tungsten-cofactor biosynthetic proteins in *Methylorubrum extorquens*.

| **Complex** | **Stoichiometry** | **ipTM** | **pTM** | **Ranking score** | **Mean pLDDT** |
| --- | --- | --- | --- | --- | --- |
| MoeA1-MobB | 2:1 | 0.67 | 0.76 | 0.69 | 85.2 |
| MoeA1-MobB-MobA | 2:1:1 | 0.52 | 0.65 | 0.55 | 81.6 |
| MoeA1-MobB-MobA-FdhD | 2:1:1:1 | 0.42 | 0.55 | 0.46 | 79.7 |
| MoeA1-FdhD | 2:1 | 0.57 | 0.69 | 0.61 | 84.4 |
| MoeA1-FdhD-MeFDH1 | 1:1:1 | 0.59 | 0.69 | 0.62 | 85.2 |
| FdhD-MeFDH1 | 1:1 | 0.74 | 0.81 | 0.76 | 88.0 |
| MoeA1-MeFDH1 | 1:1 | 0.71 | 0.79 | 0.73 | 88.6 |

# Table S3. Strains and plasmids used for gene deletion and complementation in Methylorubrum extorquens

Bacterial strains and plasmids used for gene deletion, genetic complementation, and heterologous expression of MeFDH1 and tungsten-cofactor biosynthesis components in Methylorubrum extorquens AM1.

| Reagent | Relevant properties | Source |
| --- | --- | --- |
| **Strains** | | |
| *M. extorquens* AM1 (Δ*fdh1α/β*) | Parent strain lacking *fdh1*α/β | [Jang et al. (1)](#_ENREF_1) |
| WT | *M. extorquens* AM1 (Δ*fdh1*α/β) harboring pCM110-MeFDH1 | [Jang et al. (1)](#_ENREF_1) |
| Δ*moeA1* | *moeA1* deletion in Δ*fdh1*α/β background harboring pCM110-MeFDH1 | This study |
| Δ*moeA2* | *moeA2* deletion in Δ*fdh1*α/β background harboring pCM110-MeFDH1 | This study |
| Δ*moeA3* | *moeA3* deletion in Δ*fdh1*α/β background harboring pCM110-MeFDH1 | This study |
| Δ*moeA4* | *moeA4* deletion in Δ*fdh1*α/β background harboring pCM110-MeFDH1 | This study |
| Δ*moeA5* | *moeA5* deletion in Δ*fdh1*α/β background harboring pCM110-MeFDH1 | This study |
| Δ*mog* | *mog* deletion in Δ*fdh1*α/β background harboring pCM110-MeFDH1 | This study |
| Δ*mobA-like* | *mobA-like* locus deletion in Δ*fdh1*α/β background harboring pCM110-MeFDH1 | This study |
| Δ*mobB* | *mobB* deletion in Δ*fdh1*α/β background harboring pCM110-MeFDH1 | This study |
| Δ*fdhD* | *fdhD* deletion in Δ*fdh1*α/β background harboring pCM110-MeFDH1 | This study |
| Δ*moeA1-C* | Δ*moeA1* strain complemented with *moeA1* on pCM110Duet (co-expressing MeFDH1) | This study |
| Δ*moeA1-C*(R291K) | Δ*moeA1* strain harboring pCM110-MeFDH1-MoeA1(R291K) | This study |
| Δ*moeA1-C*(A312S) | Δ*moeA1* strain harboring pCM110-MeFDH1-MoeA1(A312S) | This study |
| Δ*fdhD-C* | Δ*fdhD* strain complemented with *fdhD* on pCM110Duet (co-expressing MeFDH1) | This study |
| **Plasmids** |  |  |
| pCM184 | Allelic-exchange vector; Amp^R^ Tet^R^ Kan^R^ | [Marx and Lidstrom (2)](#_ENREF_2) |
| pCM184-*moeA1* | Knockout construct for *moeA1* in pCM184 | This study |
| pCM184-*moeA2* | Knockout construct for *moeA2* in pCM184 | This study |
| pCM184-*moeA3* | Knockout construct for *moeA3* in pCM184 | This study |
| pCM184-*moeA4* | Knockout construct for *moeA4* in pCM184 | This study |
| pCM184-*moeA5* | Knockout construct for *moeA5* in pCM184 | This study |
| pCM184-*mog* | Knockout construct for *mog* in pCM184 | This study |
| pCM184-*moaB* | Knockout construct for *moaB* in pCM184 | This study |
| pCM184-*mobA* | Knockout construct for *mobA* in pCM184 | This study |
| pCM184-*mobA*-like | Knockout construct for *mobA-*like in pCM184 | This study |
| pCM184-*mobB* | Knockout construct for *mobB* in pCM184 | This study |
| pCM184-*fdhD* | Knockout construct for *fdhD* in pCM184 | This study |
| pCM110 | Expression vector (Tet^R^) with *P_mxaF_* promoter | [Marx and Lidstrom (3)](#_ENREF_3) |
| pCM110-MeFDH1 | MeFDH1 expression plasmid (C-terminal His_6_ tag on α subunit) | [Jang et al. (1)](#_ENREF_1) |
| pCM110Duet | Duet expression vector (Tet^R^) with two *P_mxaF_* promoters and dual MCS (MCS-1, MCS-2) | This study |
| pCM110-MeFDH1-MoeA1 | pCM110Duet with MeFDH1-His (MCS-1) and *moeA1* (MCS-2) | This study |
| pCM110-MeFDH1-MoeA1(R291K) | Derivative of pCM110-MeFDH1-MoeA1 carrying the *moeA1*(R291K) substitution | This study |
| pCM110-MeFDH1-MoeA1(A312S) | Derivative of pCM110-MeFDH1-MoeA1 carrying the *moeA1*(A312S) substitution | This study |
| pCM110-MeFDH1-FdhD | pCM110Duet with MeFDH1-His (MCS-1) and *fdhD* (MCS-2) | This study |

# Table S4. Primers used in this study

Overhangs are underlined; restriction sites are shown in bold; mutated codons are indicated in lowercase. F, forward primer; R, reverse primer.

**(A)** **Cre-*loxP* knockout construction primers**

| Target (region) | Primer | Sequences (5’-3’) |
| --- | --- | --- |
| *moeA1* upstream | F | TGACGTCTAGATCT**GAATTC**CCCTGAACGAACTCGGGTTC |
|  | R | CTATACGAAGTTAT**GCGGCCGC**GTCGGTGAGCTGTGCCAT |
| *moeA1* downstream | F | CTTATCGATACCGC**GGGCCC**GGAAGCGCAGAAATTCCCAC |
|  | R | CTGGATCCTCTAGT**GAGCTC**CTCAATTTGGGAAGCGTCGC |
| *moeA2* upstream | F | TGACGTCTAGATCT**GAATTC**TGCACTCTCAATCCAGCAGG |
|  | R | ATACGAAGTTAT**GCGGCCGC**CGGGTGGGATAATTCCTCCG |
| *moeA2* downstream | F | CTTATCGATACCGC**GGGCCC**GGACGGCTTCTCCATCTAGC |
|  | R | CTGGATCCTCTAGT**GAGCTC**ACATAAACCTCGTCGCCAGG |
| *moeA3* upstream | F | GACGTCTAGATCT**GAATTC**CGTTGACGTTGCCCTTGTGTC |
|  | R | CTATACGAAGTTAT**GCGGCCGC**CTGAGACACAAACCCTCA |
| *moeA3* downstream | F | CTTATCGATACCGC**GGGCCC**CGAGATCGTCTTCGTTCGGT |
|  | R | CTGGATCCTCTAGT**GAGCTC**AAGAACTCGGTCTGCTCGAC |
| *moeA4* upstream | F | CCTGACGTCTAGATCT**GAATTC**CTGATGCGGCAGGACGAC |
|  | R | TATACGAAGTTAT**GCGGCCGC**AGGATCTTTGCGAGCGCTT |
| *moeA4* downstream | F | GCTTATCGATACCGC**GGGCCC**CTGATCCGCTCAGCCCATG |
|  | R | CTGGATCCTCTAGT**GAGCTC**GTCATGTGGAAGCGCACTTG |
| *moeA5* upstream | F | TGACGTCTAGATCT**GAATTC**CGAGATCGTCTTCGTTCGGT |
|  | R | ATACGAAGTTAT**GCGGCCGC**AAGAACTCGGTCTGCTCGAC |
| *moeA5* downstream | F | CTTATCGATACCGC**GGGCCC**CAGTGATGGTCAACCGCAAC |
|  | R | TGGATCCTCTAGT**GAGCTC**TGGACGAAGGAATCCTTGCCC |
| *mog* upstream | F | TGACGTCTAGATCT**GAATTC**CATACCTGACCACCGTCGAG |
|  | R | ATACGAAGTTAT**GCGGCCGC**CAGAGGGCATTGAGGGACTG |
| *mog* downstream | F | CTTATCGATACCGC**GGGCCC**CGTCGTTGAACGTGACATCG |
|  | R | CTGGATCCTCTAGT**GAGCTC**GAACCGGGAACTCCTTGAGG |
| *mobA*-like upstream | F | TGACGTCTAGATCT**GAATTC**CACATTTCTCAGGCGCTGGT |
|  | R | ATACGAAGTTAT**GCGGCCGC**GCTACAGGTCCGAACTGCAT |
| *mobA-like* downstream | F | CTTATCGATACCGC**GGGCCC**AGGCCTCAGCTCTTCTCTCA |
|  | R | CTGGATCCTCTAGT**GAGCTC**CTGCTCACCAAGTGCCAGAT |
| *mobB* upstream | F | TGACGTCTAGATCT**GAATTC**GCGATGTTCACCTTCCACGA |
|  | R | ATACGAAGTTAT**GCGGCCGC**AAGAACTCGGTCTGCTCGAC |
| *mobB* downstream | F | CTTATCGATACCGC**GGGCCC**GGAAGCGCAGAAATTCCCAC |
|  | R | CTGGATCCTCTAGT**GAGCTC**CTCAATTTGGGAAGCGTCGC |
| *fdhD* upstream | F | TGACGTCTAGATCT**GAATTC**TGACCATGTCGTTGTCGGTC |
|  | R | ATACGAAGTTAT**GCGGCCGC**ACAAACCATAGGCACCAGGG |
| *fdhD* downstream | F | CTTATCGATACCGC**GGGCCC**GGGCTTTCTCGTCATCACCA |
|  | R | CTGGATCCTCTAGT**GAGCTC**CAAAACCGAGCGGAACAAGG |

**(B)** **Mutant-screening primers**

| Target | Primer | Sequences (5’-3’) |
| --- | --- | --- |
| Kanamycin cassette | F1 | CGGTTTGGTTGATGCGAGTG |
|  | R1 | CATTCGTGATTGCGCCTGAG |
|  | F2 | ACACCTTCTTCACGAGGCAG |
|  | R2 | TCGCGAGCCCATTTATACCC |
| *moeA1* | F | CAGTGATGGTCAACCGCAAC |
|  | R | TTATGACGGGCTCTGCGTAC |
| *moeA2* | F | CCTGCTGTTCCCATTGGTGA |
|  | R | CCCGAACAGAACTGGCTGAA |
| *moeA3* | F | CGCGGACGCTTGAATGATG |
|  | R | CATGGCTCAGGGTCTTTCCA |
| *moeA4* | F | TGATAGACGGCTTTGAGCGG |
|  | R | ATGTTGAGCCCGGAGAGGTA |
| *moeA5* | F | GTCAGAACCGTCTCCCCTTG |
|  | R | CGACGCCGTGAACCAGAAT |
| *mog* | F1 | CTGTTGGGAGCGCATATCCT |
|  | R1 | CATCGCAATTCGCTTCCTCG |
|  | F2 | ACCAGTTCGTCTACATGGCG |
|  | R2 | TTCCCAGCGTACCTTTGTCC |
| *mobA-like* | F1 | CCCTGATCGTCACCAATCCC |
|  | R1 | GATGGCTACGAGAACTGGGG |
|  | F2 | GTAGAGGGCGAGGTCATCAC |
|  | R2 | CGACGGCATCTACGATCACA |
| *mobB* | F1 | GTCAGAACCGTCTCCCCTTG |
|  | R1 | TGCTCTCGATTTCCTACCGC |
|  | F2 | ACGATGTCCATGCCCTTCAG |
|  | R2 | TTATGACGGGCTCTGCGTAC |
| *fdhD* | F | CGAGGTGGAGCGAGAAGATC |
|  | R | TCGCCCCAATCAACCGTTTA |

**(C)** **Cloning primers**

| Target | Primer | Sequences (5’-3’) |
| --- | --- | --- |
| *moeA1* | F | AGTTGGCTGCTGCCACCGCCGGCTGGAAAGACCCTGAG |
|  | R | GTTATGCTAGTTATTGCTCACTCAGCCTCGCCGATCAG |
| *mobB* | F | GTATAAGAAGGAGATATACATGGAGGCGAATCGTGAGCG |
|  | R | GCAGCAGCCTAGGTTAATTAGAGCCCTCACCACAGCAAG |
| *fdhD* | F | GAGTTGGCTGCTGCCACCGCCCCTGGTGCCTATGGTTTGT |
|  | R | GTTATGCTAGTTATTGCTCAGAAGCTGGCAAGGAAACTGG |

**(D)** **Mutagenic primers of *moeA1***

| Target mutation | Primer | Sequences (5’-3’) |
| --- | --- | --- |
| R291K | F | GCGATCAAGCCCGGCaagCCGGTTGCGGTGGGA |
|  | R | TCCCACCGCAACCGGcttGCCGGGCTTGATCGC |
| A312S | F | CGGGCAATCCGGTGtcgGTCTACATCACG |
|  | R | CGTGATGTAGACcgaCACCGGATTGCCCG |

# Table S5. Strains and plasmids used for bacterial adenylate cyclase two-hybrid (BACTH) assays

Escherichia coli BTH101 and CyaA T25/T18 fusion plasmids used to assess pairwise protein-protein interactions by the BACTH system.

| Reagent | Relevant properties | Source |  |
| --- | --- | --- | --- |
| **Strain** | | | |
| BTH101 | F^-^ *cya-99, araD139, galE15, galK16, rpsL1 (Str r), hsdR2, mcrA1, mcrB1* | Euromedex |  |
| **Plasmids** | | | |
| pKT25 | pACYC184-derived vector carrying the CyaA(T25) fragment under *lac* promoter control; Kan^R^ | Euromedex |  |
| pUT18 | pUC19-derived vector carrying the CyaA(T18) fragment under *lac* promoter control; Amp^R^ | Euromedex |  |
| pKT25-zip | Positive-control plasmid expressing T25-GCN4 leucine zipper (strong interaction control); Kan^R^ | Euromedex |  |
| pUT18-zip | Positive-control plasmid expressing GCN4 leucine zipper-T18 (strong interaction control); Amp^R^ | Euromedex |  |
| pKT25-MoeA1 | pKT25 expressing an N-terminal T25-MoeA1 fusion (*lac* promoter); Kan^R^ | This study |  |
| pKT25-MobA | pKT25 expressing an N-terminal T25-MobA fusion (*lac* promoter); Kan^R^ | This study |  |
| pKT25-MobB | pKT25 expressing an N-terminal T25-MobB fusion (*lac* promoter); Kan^R^ | This study |  |
| pKT25-MoaB | pKT25 expressing an N-terminal T25-MoaB fusion (*lac* promoter); Kan^R^ | This study |  |
| pKT25-FdhD | pKT25 expressing an N-terminal T25-FdhD fusion (*lac* promoter); Kan^R^ | This study |  |
| pUT18-MoeA1 | pUT18 expressing a C-terminal MoeA1-T18 fusion (*lac* promoter); Amp^R^ | This study |  |
| pUT18-MobA | pUT18 expressing a C-terminal MobA-T18 fusion (*lac* promoter); Amp^R^ | This study |  |
| pUT18-MobB | pUT18 expressing a C-terminal MobB-T18 fusion (*lac* promoter); Amp^R^ | This study |  |
| pUT18-MoaB | pUT18 expressing a C-terminal MoaB-T18 fusion (*lac* promoter); Amp^R^ | This study |  |
| pUT18-FdhD | pUT18 expressing a C-terminal FdhD-T18 fusion (*lac* promoter); Amp^R^ | This study |  |

# Table S6. Representative molybdate insertases and tungsten-associated MoeA homologues

| **Organism** | **Protein name** | **Accession number** | **References** |
| --- | --- | --- | --- |
| **W-specific MoeA homologues** | | | |
| *Methylorubrum extorquens* | MoeA1 | C5AVW2 | [Laukel et al. (4)](#_ENREF_4) |
| *Aromatoleum aromaticum* | MoeA | Q5P7M0 | [Winiarska et al. (5)](#_ENREF_5) |
| *Geobacter metallireducens* | MoeA | Q39YU5 | [Huwiler et al. (6)](#_ENREF_6) |
| *Pyrococcus furiosus* | MoeA | Q8U034 | [George et al. (7)](#_ENREF_7) |
| *Campylobacter jejuni* | MoeA | Q0PA33 | [Smart et al. (8)](#_ENREF_8) |
| *Syntrophobacter fumaroxidans* | MoeA | A0LMU7 | [de Bok et al. (9)](#_ENREF_9) |
| *Methanocaldococcus jannaschii* | MoeA | Q58080 | [Bult et al. (10)](#_ENREF_10) |
| *Eubacterium acidaminophilum* | MoeA | W8T1Y5 | [Makdessi et al. (11)](#_ENREF_11) |
| *Desulfovibrio alaskensis* | MoeA | Q316W5 | [Otrelo-Cardoso et al. (12)](#_ENREF_12) |
| *Methanococcus maripaludis* | MoeA | Q6LWT7 | [Costa et al. (13)](#_ENREF_13) |
| **Classical Mo-insertases** | | | |
| *Arabidopsis thaliana* | CNX1 | Q39054 | [Krausze et al. (14)](#_ENREF_14) |
| *Rattus norvegicus* | GephE | Q03555 | [Kasaragod and Schindelin (15)](#_ENREF_15) |
| *Drosophila melanogaster* | Cinnamon | P39205 | [Kamdar et al. (16)](#_ENREF_16) |
| *Escherichia coli* | MoeA | P12281 | [Xiang et al. (17)](#_ENREF_17) |

# References

1. Jang, J., Jeon, B. W., and Kim, Y. H. (2018) Bioelectrochemical conversion of CO2 to value added product formate using engineered Methylobacterium extorquens. *Scientific Reports* **8**, 7211

2. Marx, C. J., and Lidstrom, M. E. (2002) Broad-Host-Range cre-lox System for antibiotic marker recycling in gram-negative bacteria. *BioTechniques* **33**, 1062-1067

3. Marx, C. J., and Lidstrom, M. E. (2001) Development of improved versatile broad-host-range vectors for use in methylotrophs and other Gram-negative bacteria. *Microbiology* **147**, 2065-2075

4. Laukel, M., Chistoserdova, L., Lidstrom, M. E., and Vorholt, J. A. (2003) The tungsten-containing formate dehydrogenase from Methylobacterium extorquens AM1: Purification and properties. *European Journal of Biochemistry* **270**, 325-333

5. Winiarska, A., Ramírez-Amador, F., Hege, D., Gemmecker, Y., Prinz, S., Hochberg, G. *et al.* (2023) A bacterial tungsten-containing aldehyde oxidoreductase forms an enzymatic decorated protein nanowire. *Science Advances* **9**, eadg6689

6. Huwiler, S. G., Löffler, C., Anselmann, S. E. L., Stärk, H.-J., von Bergen, M., Flechsler, J. *et al.* (2019) One-megadalton metalloenzyme complex in Geobacter metallireducens involved in benzene ring reduction beyond the biological redox window. *Proceedings of the National Academy of Sciences* **116**, 2259-2264

7. George, G. N., Prince, R. C., Mukund, S., and Adams, M. W. W. (1992) Aldehyde ferredoxin oxidoreductase from the hyperthermophilic archaebacterium Pyrococcus furiosus contains a tungsten oxo-thiolate center. *Journal of the American Chemical Society* **114**, 3521-3523

8. Smart, J. P., Cliff, M. J., and Kelly, D. J. (2009) A role for tungsten in the biology of Campylobacter jejuni: tungstate stimulates formate dehydrogenase activity and is transported via an ultra-high affinity ABC system distinct from the molybdate transporter. *Molecular Microbiology* **74**, 742-757

9. de Bok, F. A. M., Hagedoorn, P.-L., Silva, P. J., Hagen, W. R., Schiltz, E., Fritsche, K. *et al.* (2003) Two W-containing formate dehydrogenases (CO2-reductases) involved in syntrophic propionate oxidation by Syntrophobacter fumaroxidans. *European Journal of Biochemistry* **270**, 2476-2485

10. Bult, C. J., White, O., Olsen, G. J., Zhou, L., Fleischmann, R. D., Sutton, G. G. *et al.* (1996) Complete Genome Sequence of the Methanogenic Archaeon, Methanococcus jannaschii. *Science* **273**, 1058-1073

11. Makdessi, K., Andreesen, J. R., and Pich, A. (2001) Tungstate Uptake by a Highly Specific ABC Transporter inEubacterium acidaminophilum. *Journal of Biological Chemistry* **276**, 24557-24564

12. Otrelo-Cardoso, A. R., Nair, R. R., Correia, M. A. S., Rivas, M. G., and Santos-Silva, T. (2014) TupA: A Tungstate Binding Protein in the Periplasm of Desulfovibrio alaskensis G20. *International Journal of Molecular Sciences* **15**, 11783-11798

13. Costa, K. C., Wong, P. M., Wang, T., Lie, T. J., Dodsworth, J. A., Swanson, I. *et al.* (2010) Protein complexing in a methanogen suggests electron bifurcation and electron delivery from formate to heterodisulfide reductase. *Proceedings of the National Academy of Sciences* **107**, 11050-11055

14. Krausze, J., Hercher, T. W., Zwerschke, D., Kirk, M. L., Blankenfeldt, W., Mendel, R. R. *et al.* (2018) The functional principle of eukaryotic molybdenum insertases. *Biochemical Journal* **475**, 1739-1753

15. Kasaragod, Vikram B., and Schindelin, H. (2016) Structural Framework for Metal Incorporation during Molybdenum Cofactor Biosynthesis. *Structure* **24**, 782-788

16. Kamdar, K. P., Shelton, M. E., and Finnerty, V. (1994) The Drosophila molybdenum cofactor gene cinnamon is homologous to three Escherichia coli cofactor proteins and to the rat protein gephyrin. *Genetics* **137**, 791-801

17. Xiang, S., Nichols, J., Rajagopalan, K. V., and Schindelin, H. (2001) The Crystal Structure of *Escherichia coli* MoeA and Its Relationship to the Multifunctional Protein Gephyrin. *Structure* **9**, 299-310
